# Tissue-enhanced plasma proteomic analysis for disease stratification in amyotrophic lateral sclerosis

**DOI:** 10.1186/s13024-018-0292-2

**Published:** 2018-11-07

**Authors:** Irene Zubiri, Vittoria Lombardi, Michael Bremang, Vikram Mitra, Giovanni Nardo, Rocco Adiutori, Ching-Hua Lu, Emanuela Leoni, Ping Yip, Ozlem Yildiz, Malcolm Ward, Linda Greensmith, Caterina Bendotti, Ian Pike, Andrea Malaspina

**Affiliations:** 1 Neuroscience and Trauma Centre, Blizard Institute, Barts and The School of Medicine and Dentistry, Queen Mary University of London, 4 Newark Street, London, City of London, Greater London E1 2AT UK; 2 Proteome Sciences plc, Hamilton House, Mabledon Place, London, UK; 3 Laboratory of Molecular Neurobiology, Department of Neuroscience, IRCCS - Istituto di Ricerche Farmacologiche Mario Negri, Milan, Italy; 4 Sobell Department of Motor Neuroscience and Movement Disorders, MRC Centre for Neuromuscular Disorders, UCL Institute of Neurology, University College London, London, UK; 5 Department of Neurology, China Medical University Hospital, Taichung City, Taiwan

**Keywords:** Amyotrophic lateral sclerosis, Proteomics, Biomarkers, TMTcalibrator™, SOD1G93A animal models

## Abstract

**Background:**

It is unclear to what extent pre-clinical studies in genetically homogeneous animal models of amyotrophic lateral sclerosis (ALS), an invariably fatal neurodegenerative disorder, can be informative of human pathology. The disease modifying effects in animal models of most therapeutic compounds have not been reproduced in patients. To advance therapeutics in ALS, we need easily accessible disease biomarkers which can discriminate across the phenotypic variants observed in ALS patients and can bridge animal and human pathology. Peripheral blood mononuclear cells alterations reflect the rate of progression of the disease representing an ideal biological substrate for biomarkers discovery.

**Methods:**

We have applied TMTcalibrator™, a novel tissue-enhanced bio fluid mass spectrometry technique, to study the plasma proteome in ALS, using peripheral blood mononuclear cells as tissue calibrator. We have tested slow and fast progressing SOD1G93A mouse models of ALS at a pre-symptomatic and symptomatic stage in parallel with fast and slow progressing ALS patients at an early and late stage of the disease. Immunoassays were used to retest the expression of relevant protein candidates.

**Results:**

The biological features differentiating fast from slow progressing mouse model plasma proteomes were different from those identified in human pathology, with only processes encompassing membrane trafficking with translocation of GLUT4, innate immunity, acute phase response and cytoskeleton organization showing enrichment in both species. Biological processes associated with senescence, RNA processing, cell stress and metabolism, major histocompatibility complex-II linked immune-reactivity and apoptosis (early stage) were enriched specifically in fast progressing ALS patients. Immunodetection confirmed regulation of the immunosenescence markers Galectin-3, Integrin beta 3 and Transforming growth factor beta-1 in plasma from pre-symptomatic and symptomatic transgenic animals while Apolipoprotein E differential plasma expression provided a good separation between fast and slow progressing ALS patients.

**Conclusions:**

These findings implicate immunosenescence and metabolism as novel targets for biomarkers and therapeutic discovery and suggest immunomodulation as an early intervention. The variance observed in the plasma proteomes may depend on different biological patterns of disease progression in human and animal model.

**Electronic supplementary material:**

The online version of this article (10.1186/s13024-018-0292-2) contains supplementary material, which is available to authorized users.

## Background

Amyotrophic lateral sclerosis (ALS), a fatal neurodegenerative disorder, is a clinically heterogeneous condition where survival can be less than a year or more than a decade from symptoms onset, making any assessment of treatment response in clinical trials difficult in absence of reliable estimates of prognosis [1]. A significant diagnostic delay in ALS prevents early treatment reducing the prospect of therapeutic success [2]. Mutant superoxide dismutase 1 (SOD1G93A) transgenic mice models of ALS are widely used as surrogates of human pathology in pre-clinical research. A relatively uniform genetic background and the strictly controlled environmental and breeding conditions make SOD1G93A transgenic animals ideal models to investigate the disease pathobiology and the pre-symptomatic disease stage [3, 4].

Experimental evidence suggests that the rate of disease progression in ALS may be linked to the immunological response to neuronal degeneration, which is reproduced systemically by altered blood levels of cytokines, acute phase reactants and by an early down-regulation of FoxP3-positive T regulatory cells predominantly in patients with a faster disease progression [5, 6]. In SOD1G93A transgenic mouse models of ALS, symptomatic disease can develop with a slow and fast progression in C57 and 129Sv genetic backgrounds respectively (here defined as Sym-SOD1C57 and Sym-SOD1129S). In these SOD1G93A transgenic mice, impairment of the protein quality control in the spinal cord and the activation of the major histocompatibility complex I (MHCI) expression in axons and neuromuscular junctions are considered indices of fast disease progression [7, 8].

The inflammatory response in ALS co-exists with a state of deranged lipid metabolism and altered total daily energy expenditure [6, 9, 10], associated also with the effects of genetic mutations of the TDP-43 and C9orf72 genes linked to familial ALS [11–14]. Importantly, it is also acknowledged that functionally competent and high energy-demanding motor neurons are vulnerable to chronic inflammation and changes in metabolism [15–17] while aging, one of the main risk factors for ALS, may increase neuronal vulnerability with a decline in cell glucose uptake and mitochondrial energy production [18].

Over 50 randomized controlled clinical trials in ALS have been unsuccessful, despite many compounds having shown a disease-modifying effect in animal models [19–21]. There is therefore a critical need to improve translation of pre-clinical results into clinical trial outcomes in ALS, through the identification of molecular factors driving disease progression in humans and rodents which can be used for phenotypic stratification across species. To this end, a viable strategy is the molecular profiling of accessible biofluids and of Peripheral Blood Mononuclear Cells (PBMC), central to many aspects of the systemic immunological reaction to neurodegeneration [22, 23]. TMTcalibrator™, an unbiased proteomics method, is ideally placed to combine tissue and fluid proteomics in a single experiment, providing significant gains in sensitivity and a direct link between protein expression in diseased tissue and in matched fluids [24].

In this study, we have used TMTcalibrator™ to investigate biological features which are regulated in plasma and are also coherently expressed in matched PBMC samples from fast and slow progressing ALS patients (defined as ALS-Fast and ALS-Slow), at an early and late stage of the disease, and used immunoassays to re-test the plasma expression of the same markers in a more extended cohort of similarly stratified ALS patients. We have used the same approach to test pre-symptomatic and symptomatic SOD1G93A transgenic mouse models of ALS with a fast (129Sv strain) and a slow (C57 strain) progression of the disease. We have detected an early inflammatory and acute phase response in both human and animal model, while perturbed metabolism and an overall switch to cellular senescence is seen in fast progressing human pathology. We found only a partial overlap of the plasma/PBMC proteome between humans and rodents, suggesting a variability which is disease-stage dependent.

## Methods

### Selection of patients

ALS patients and biological samples were selected from the ALS biomarkers study biorepository (Ethical approval: London and the City Research Ethics Committee 1 09/H0703/27). Exclusion criteria were neuroinflammatory and neurodegenerative comorbidities, recent mechanical injuries and/or infections, systemic autoimmune disorders, cancer, pro-thrombotic states, family history of ALS or frontotemporal dementia (FTD) as well as known genetic mutation linked to ALS or FTD. The Functional Rating Scale-Revised (ALSFRS-R; range 1 to 48, with increasing levels of neurological impairment with lower scores) was used to define the level of neurological impairment, with early ALS corresponding to a score > 40 and late ALS < 35. Disease progression to last visit (PRL) was calculated as “48 - ALSFRS-R at the last visit, divided for disease duration from onset to the last visit in months” (fast ALS progression: PRL > 0.7; slow ALS progression: PRL < 0.5) [6] .

### Animal models

Female superoxide dismutase 1 (SOD1) transgenic mice with a G93A mutation in a C57BL/6JOlaHsd (C57SOD1G93A) and in a 129SvHsd (129SvSOD1G93A) genetic background and wild-type female littermates (defined as WTC57 and WT129Sv) were used in this study (Jackson Laboratories, B6SJL-TgNSOD-1-SOD1G93A-1Gur). The development of the symptomatic stage of the disease differed significantly in the two SOD1G93A transgenic mouse strains, with regards to disease onset and duration [8, 25]. In this study, fast developing symptomatic SOD1G93A transgenic mice are defined as Sym-SOD1129Sv (129Sv pre-symptomatic mice as Pre-SOD1129Sv), while slow symptomatic progressing mice as Sym-SOD1C57 (C57 pre-symptomatic mice as Pre-SOD1C57). SOD1G93A transgenic animals expressed 20 copies of the human Gly93Ala SOD1 gene substitution in the C57OlaHsd and 129SvHsd backgrounds for more than 30 and 10 generations respectively. Mice were maintained in a specific pathogen-free environment (21 °C temperature, 10% relative humidity in a 12 h of light/dark cycle) with food/water supplied ad libitum and adaptations for animals with a substantial motor impairment. Mice of both strains were considered in a symptomatic stage when they exhibited a 50% decrease in latency of grip strength and a 45% body weight decline from the peak values in the pre-symptomatic stage [26]. Plasma samples from Wild type (WT) animals used in the re-test experiments were obtained at the same time of blood collection from both pre-symptomatic and symptomatic SOD1G93A transgenic animal models.

### Sample collection, plasma and mononuclear cell extraction and processing

18 ml blood were collected in EDTA tubes and centrifuged at 800 g for 10 min at room temperature. Plasma was recovered and centrifuged at 3500 rpm for 10 min and stored at − 80 °C. Plasma samples were albumin and IgG depleted (ProteoPrep® Immunoaffinity kit). PBMC were isolated by density-gradient 2000 rpm centrifugation for 40 min at 20 °C using Lymphoprep™ (Alere) and subsequently washed in Dulbecco’s phosphate-buffered saline (Gibco). PBMC pellets were stored at − 80 °C in a freezing solution (10% DMSO in foetal bovine serum). After 24 h, PBMC aliquots were transferred into liquid nitrogen. PBMC samples were thawed at 37 °C and re-suspended in 10 ml warm (37 °C) Dulbecco phosphate buffer. Cell suspension was centrifuged at room temperature and the pellet was re-suspended in 100 μl of lysis buffer (8 M urea, 75 mM NaCl, 50 mM Tris, pH 8.2, protease inhibitors cocktail, cOmplete™, Mini Protease Inhibitor Co, Sigma). Blood was collected from mice cheek and diluted in 3 ml medium (2.5 mM EDTA, 2% FBS in PBS) for PBMC and plasma fractions isolation as reported above. PBMC samples were lysed, sonicated and stored at − 80 °C.

### In-solution tryptic digest, tandem mass tag (TMT®) labelling and TMT calibrator™

Protein quantification was carried out using a Bradford Protein Assay (Bio Rad). 100 μg (30 μg for mice) protein from depleted plasma and 1 mg (750 μg for mice) from the PBMC pool were dried down in a vacuum centrifuge (SpeedVac, Thermo Scientific). Following solubilisation and denaturation in 100 mM Triethylammonium bicarbonate (TEAB)/0.1% (*w*/*v*) SDS), samples were reduced with 1 mM tris (2-carboxyethyl) phosphine 10 (TCEP) at 55 °C for 60 min and alkylated with 7.5 mM iodoacetamide at room temperature (RT) for 60 min. Trypsin (MS grade, Promega) was added at a 1:25 (*w*/w) ratio to total protein and incubated at 37 °C overnight. Digestion products were labelled with TMT®10plex reagents (Thermo Scientific) and incubated at RT for 60 min. Six of the 10 isobaric TMT® reagents were used to label individual plasma samples and the total amount of PBMC protein pool was divided among the remaining 4 TMT channels (the four-point calibrator) comprising 1/21, 4/21, 6/21 and 10/21 total protein respectively. To quench the TMT® reaction, 0.25% hydroxylamine was added. The samples were then combined to form the analytical 10plex sets, desalted in RP18 columns and dried under vacuum. The human plasma samples were divided into four TMT®10plex sets, two for the early and two for the late time point plasma samples. Each set contained three ALS-Fast and three ALS-Slow plasma samples in combination with the four-point PBMC pool lysate (human PBMC representing all the conditions and phenotypic variants under investigation) used as calibrator (Table 1B). The same experimental design was applied to the animal model study, where four different TMT®10plex sets were used to compare 24 samples in total. Two TMT®10plex sets included samples from transgenic mice, one for Pre-SOD1129Sv and Pre-SOD1C57, and the other one for Sym-SOD1129Sv and Sym-SOD1C57 (*n* = 3 for each genetic background plus 4 channels in each set for the mouse PBMC pool calibrator). The remaining two TMT®10plex sets included plasma samples from the equivalent Wild type (WT) animals for both 129Sv and C57genetic backgrounds (n = 3 per group, plus 4 channels in each set for the mouse PBMC pool calibrator), collected at the same time blood was taken from pre-symptomatic and symptomatic animal (Additional file 1: Figure S1 A, shows sample distribution across TMT®10plexes).

**Table 1:** Demographics and clinical characteristics of ALS (A,B,C) and healthy control (D) individuals included in the discovery proteomics (A, B) and in the re-test immunoassays (C, D)

A. DISCOVERY EXPERIMENT: ALS PLASMA SAMPLES									
ALS type	ALSFRS-R early	ALSFRS-R late	Gender M/F	Age at onset (years)	PRL	Time from onset to death or last visit (months)	Time from onset to first sample (months)	Time between first and last sample (months)	Site of disease onset limb/ bubar
*Slow (n = 6)*	43 (41–45)	31.5 (27–35)	5 M / 1F	58.1 (35–71)	0.25 (0.1–0.4)	107 (97, 254)	76 (30–192)	58 (48–69)	4 limb /2 bulbar
*Fast* *(n = 6)*	43.8 (41–46)	28.6 (22–35)	3 M / 3F	61.3 (48–67)	1.5 (1–1.9)	20.5 (11–32)	10.2 (5–19)	13.8 (7–22)	3 limb /3 bulbar
B. DISCOVERY EXPERIMENT: ALS PBMC SAMPLE TO FORM THE REFERENCE POOL									
ALS Pool	ALSRS-R	Gender M/F		Age at onset (years)	PRL	Time from onset to death or last visit (months)	Time from onset to sampling (months)		Site of disease onset limb/ bubar
*Slow(*3) *Fast* (2) *n* = 5	31 (22–42)	3 M /2 F		65.8 (57–68)	0.8 (0.02–2.5)	64.6 (8–120)	19 (7–44)		3 limb /2 bulbar
C. RE-TEST EXPERIMENT: ALS PLASMA SAMPLES									
ALS type	ALSRS-R	Gender M/F		Age at onset (years)	PRL	Time from onset to death or last visit (months)	Time from onset to sampling (months)		Site of disease onset limb/ bubar
*Slow* (*n* = 23)	39 (18–47)	18 M / 5F		65.25 (37–86.9)	0.216 (0.03–0.47)	120.4 (28–335.2)	62.8 (7–248.8)		15 limb /8 bulbar
*Fast* (*n* = 24)	33 (13–43)	8 M / 16F		62.8 (34.8–82.4)	1.48 (0.74–3.6)	28.1 (5–50)	14.8 (2.8–29.8)		13 limb /11 bulbar
D. RE-TEST EXPERIMENT: HEALTHY CONTROLS PLASMA SAMPLES									
*n* = 29	Gender M/F					Age at sampling (years)			
	8 M / 17F					60.9 (50.8–73)			

### Strong cation exchange (SCX) fractionation and liquid chromatography tandem mass spectrometry (LC-MS/MS)

Each analytical 10plex sample was reconstituted and loaded onto a Polysulfoethyl-A column (4.6 × 100 mm, 5 μm, 200 Å, PolyLC) attached to a Waters 2695 HPLC. Quantitative analysis was performed using an Orbitrap Fusion™ Tribrid™ mass spectrometer in positive ion mode with an EASY nLC1000 system and 50 cm EASY-Spray column (all Thermo Scientific). More details on the SCX and LC-MS/MS methodologies are reported in the Additional file 2.

### Mass spectrometry analysis and computational proteomics

All plasma samples and pooled PBMC lysates used as tissue calibrator passed sample quality control protocols (data not shown) and were divided into four TMT®10plex sets. All mass spectrometry files were inspected independently and passed internal quality control metrics (data not shown). Outputs of computational proteomics from the 40 raw data files from each TMT®10plex study (human and animal model study) were assembled into a single dataset and processed by Proteome Sciences’ proprietary workflows for TMTcalibrator™ including data integration (CalDIT), pre-processing and feature selection (FeaST). Normalised quantitative data were provided for all plasma peptides channels as a ratio against the calculated mid-point value of the PBMC calibrator channels (see Additional file 1: Figure S1 describing the experimental workflow).

### Peptide identification and quantification

For each experiment, raw mass spectrometry data files were submitted to Proteome Discoverer (PD) v1.4 (Thermo Scientific), using the Spectrum Files node. The spectrum selector was set to its default values while the SEQUEST-HT node was set to search data against the human or mouse (supplemented with the human SOD1 sequence) FASTA UniProtKB and Swiss-Prot database respectively. A more detailed description of the peptide identification and quantification process is reported in Additional file 2.

### Data assembly, pre-processing and normalisation

In the first step of the CalDIT workflow, reporter ion intensities were corrected to remove the contribution of signals from adjacent reporter ion channels. Subsequently, within each mass spectrometry run, intensity values across the four calibrant channels (129C, 130 N, 130C, 131) were normalised (median-scaling) and, for each peptide spectrum match (PSM), a reference intensity value was calculated based on the calibrant intensity distribution. This reference value was then used to calculate Log_2_-transformed PSM ratio values for each of the experimental channels, resulting in lower variance between PSM-level quantifications of the same peptide sequence at different points of the elution profile, or MS runs. To generate peptide expression (ratio) values, median values were then computed across quantified PSMs of the same peptide sequence.

In the first step of the FeaST workflow, peptides with more than ~ 35% missing quantitative values within an experimental group (i.e. ALS-Fast-Early; ALS-Fast-Late; ALS-Slow-Early; ALS-Slow-Late) were removed from subsequent analysis. Remaining peptides, with values below the percentage threshold, were replaced by values imputed using the k-nearest neighbours (*n* = 2) imputation method, applied to samples within each experimental group. In order to reduce the batch effect created by the use of multiple TMT®10 plexes, a LIMMA-based batch effect correction procedure was applied (Additional file 1: Figure S2), using a linear model constructed on the TMT® 10plex batch number and TMT® channel and specifying the experimental groups (PCA before batch effect correction Additional file 1: Figure S2). For protein-level analysis, the same procedure was applied in a parallel analysis and, subsequently, expression values were computed by averaging (trimmed mean, trim factor: 0.2) ratios of all non-phosphorylated peptides which matched uniquely to the gene identifier.

Two quality control metrics per sample were calculated: the median (measure of central tendency) and the inter-quartile range (IQR), measure of scale, using peptide and protein distributions. A sample was considered as a strong outlier if either QC metric value was more than three standard deviations from the overall mean.

### Immunoassays

Expression analysis of 5 protein candidates in mouse and human plasma samples was undertaken by enzyme-linked immunosorbent assay (ELISA) using commercial kits, a electrochemiluminescence (ECL)-based Meso Scale Discovery (MSD) platform and by Western blot. Plasma samples were processed, aliquoted and frozen at − 80 °C within 1 h from blood collection, according to standard consensus procedures. ECL-MSD was used to quantify Apolipoprotein E (APOE; R-PLEX Antibody Set F212I), Galectine-3 (R-PLEX Antibody Set F214F) and transforming growth factor beta-1 (TGFB1; U-PLEX kit K151XWK), in human and mouse plasma, and Apolipoprotein A1 (ApoA1; R-PLEX Antibody Set F21PR) in human. A commercial ELISA was used to analyse ITGB3 and Apo A1 in mouse plasma (Biorbyt orb408222 and Abbexa abx254777 respectively) as well ITGB3 in human plasma (Biorbyt orb407522). Standards, primary and secondary antibodies, detection range including lower and upper limits of detection were specified in the manufacturer’s conditions. Plasma samples from Wild-type (WT) and SOD1G93A transgenic mice (both pre-symptomatic and symptomatic), from ALS-Fast, ALS-Slow and healthy controls were equally distributed on each plate and measured in duplicate. Each plate contained a target-specific calibrator: Apolipoprotein A1 (APOA1: 0–1,000,000 pg/ml for human and 7.81-500 ng/ml for mouse); Apolipoprotein E (APOE: 0–200,000 pg/ml); Galectin-3 (03,000 pg/ml), TGFB1 (0–56,600 pg/ml) and ITGB3 (125–8000 pg/ml for human 62.5-4000 pg/ml for mice).

For Western blot analysis of ITGB3, 40 μg of albumin-depleted proteins were diluted in Laemmli buffer and loaded onto 10% acrylamide gels. After electrophoresis, proteins were transferred onto nitrocellulose membranes and blocked with Tris buffered saline 0.1% Tween (TBS-T) containing 5% non-fat dry milk powder and − 20 for 1 h at room temperature. Membranes were then incubated overnight with rabbit anti-Integrin β3 antibody 1:1000 (Integrin β3, D7X3P XP® Rabbit mAb #13166. Cell Signaling Technology, Inc.) and with anti-Galectin-3 antibody (Mouse monoclonal Galectin 3 ab2785, Abcam Ltd.) in TBS-T (0.1%) containing 5% bovine serum albumin (BSA) and further incubated with horseradish peroxidase (HRP)-conjugated swine anti-rabbit 1:2500 (Dako) as secondary antibody in TBS-T (0.1%) containing 5% BSA. Enhanced chemiluminescence (ECL kit; GE Healthcare), the ChemiDoc XRS+ imaging system and the image lab 5.2.1 (Bio-Rad) software were used for signal detection acquisition and analysis.

### Statistical analysis and data mining

All statistical methods applied for the proteomic data analysis were performed using an in-house workflow called FeaST, developed in R statistical programming environment. Principal component analysis **(**PCA) score and loading plots were generated to study the variance structure of the data sets, indicating technical and biological factors and the influence of each step in the data pre-processing and normalisation workflow. Multifactorial linear modelling (LIMMA) was applied to determine significantly regulated features (peptides or proteins), using the following linear model: logRatio ~ Class + Age + Stage + Group (ALS/Control). Log_2_ fold changes (logFC) and *p*-values were calculated for all peptides and proteins that passed the filtering criteria described (above). The significance criterion α was standardly set to 0.05 to consider a feature as “regulated”. Multiple testing was performed using a Benjamini-Hochberg correction.

Proteome Sciences’ proprietary workflows for functional analysis (FAT) were used to identify differences among phenotypic variants in biological processes extracted from plasma proteomes. Significance of *enrichment* was evaluated based on the results of Fisher’s Exact Test and multiple test corrections were applied (Benjamini-Hochberg). Functional terms used in this analysis included Gene Ontology Biological Processes and Reactome Pathways. Human and mouse-specific annotations were extracted from publicly-available data repositories. A minimum of two matched gene names was required and terms were considered significant for a 3-group comparison.

For the immunoassay data, statistical analysis was performed using GraphPad Prism 6. Continuous variables were presented in median (interquartile range) and nonparametric analysis for group comparisons (with Dunn’s multiple comparisons test) as well as correlation analysis were applied. We used log rank analysis (Mantel-Cox test) to compare survival (fixed date was used to censor data for survival analysis). Receiver operating characteristic curve analysis was used to assess assay sensitivity/specificity and diagnostic performance. A *p* value of less than 0.05 was considered statistically significant.

We have used a RNA-Seq transcriptome and splicing database of glia, neurons and vascular cells of the cerebral cortex to look at cell type-specific expression in the central nervous system (CNS) of Galectin-3, TGFb1 and ITGB3 (http://www.brainrnaseq.org/).

## Results

### Patients and controls

17 patients with a diagnosis of possible, probable, laboratory-supported and definite ALS according to the El Escorial criteria [27] were enrolled in the discovery study (demographic and clinical features reported in Table 1A, B). Plasma samples from 12 of these, including six ALS-Fast (progression rate to last visit (PRL) > 0.7; male/female ratio 3/3, average age at disease onset 61.7, (48–67; average disease duration to death or last visit 20.5 months (11–32)) and six with a slow rate of progression (PRL < 0.5; 5 male/1 female, average age at disease onset 58.1, 35–71; average disease duration to death or last visit 107 months (97, 254)) were included in the exploratory proteomics as analytical samples, while PBMC samples isolated from blood donated by the remaining five patients were used in the tissue calibrant channels (PRL range: 0.023–2.5; three male / two female, average age at disease onset 65.8, 57–68), (Table 1).

Blood samples from an additional cohort of 47 ALS patients, including 24 ALS-Fast and 23 ALS-Slow, and 29 healthy age and gender-matched controls were used to re-test plasma expression of selected protein candidates using immunodetection. The average age of disease onset among the ALS sub-groups was comparable to the average age of sampling in the healthy control group (Table 1C, D). The most common genetic mutations linked to familial ALS were excluded and all ALS patients had plasma CRP and ferritin levels within normal limits at the time of sampling (normal values: CRP < 5 mg/L; ferritin 10–160 μg/L) [6].

The ALS functional rating scale revised (ALSFRS-R; 1 to 48, higher level of neurological disability with lower score) was used to define early (ALSFRS-*R* ≥ 40) and late (ALSFRS-*R* ≤ 35) time points in the ALS cohort used for the discovery experiment. ALS patients were stratified according to disease progression rate to last visit (PRL: 48 minus the ALSFRS-R score at the last visit, divided per disease duration from onset in months) in slow (PRL < 0.5) and fast (PRL > 0.7). PBMC samples were extracted from blood taken from 5 ALS patients with a variable rate of disease progression (B). Age at onset and gender for both patients and healthy matched controls are also reported. All individuals belonged to the same ethnic group (Caucasian; except for one patients who was of Asian ethnicity). Duration of the disease from onset of first symptoms (e.g. weakness or speech impairment) to both sampling time and death/last visit is reported. For each sub-group, values are reported as mean (average).

### Exploratory analysis using the feature selection tool (FeaST)

**Fig. 1:**
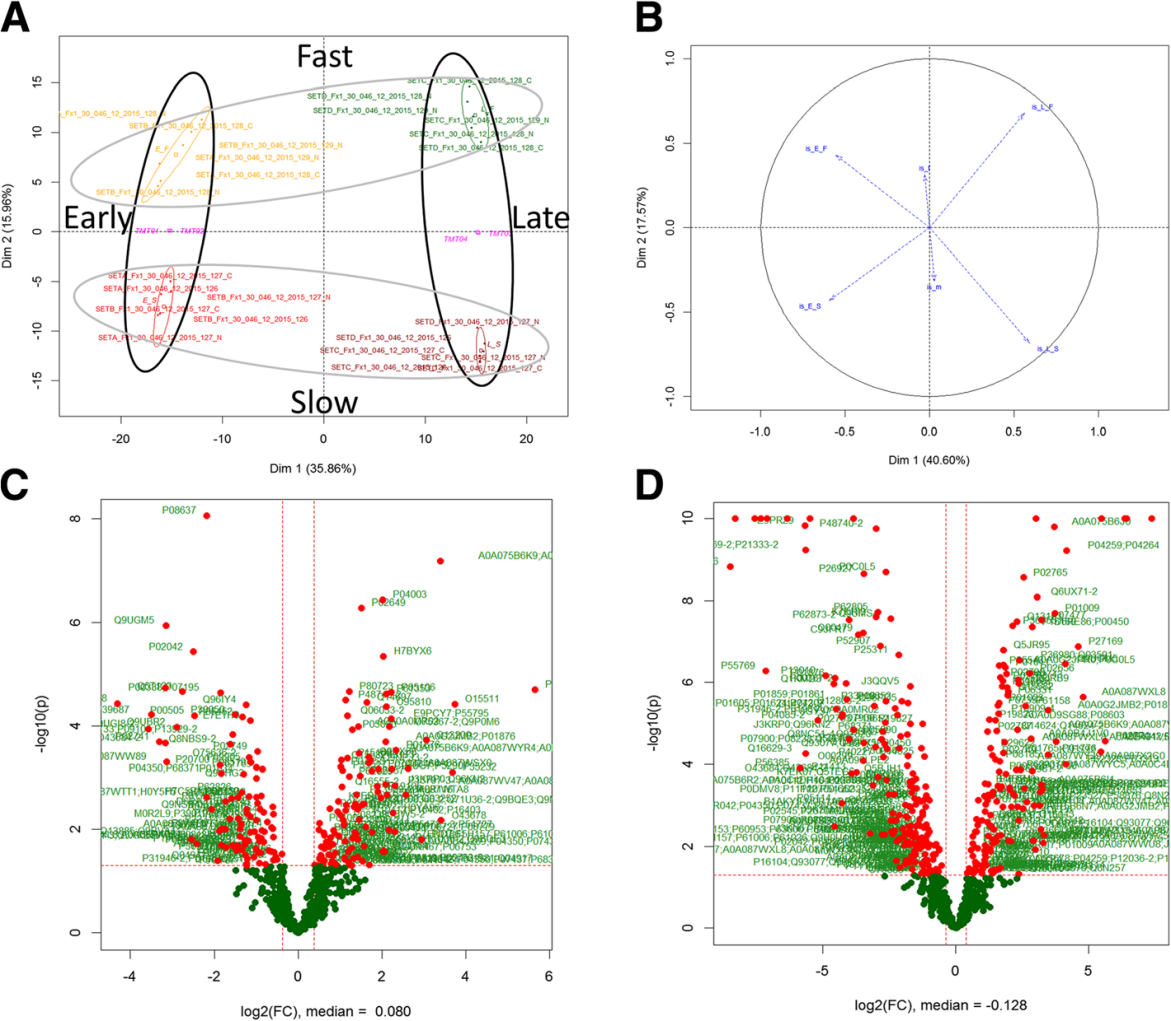
Distribution of differentially regulated features and ALS patient phenotypic variants: (**a**) Principal component analysis (PCA) using scores plots before feature selection (colour codes: fast-early: yellow; fast-late: green; slow-early: red; slow-late: brown). The disease stage dimension is the main contributor to the separation in the first component (35.86%) and the rate of disease progression is the main contributor to the separation on the second component (15%). **b** PCA loadings plot generated with data derived from regulated features shows a more significant separation in the first compared to second component (40.6% and 17.5% respectively). **c** Volcano plot showing significantly regulated features comparing fast and slow progressing ALS patients in the early time point (cross-sectional study). **d** Volcano plot showing significantly regulated features comparing early and late time points for the slow progressing ALS patients (longitudinal study). Volcano plots agree with PCA showing a more significant difference between early and late time points when compared to fast versus slow disease progression

Differentially regulated plasma protein signatures across the four phenotypic variants were analysed using principal component analysis (PCA), as illustrated in Fig. 1a. The strongest driver of variance in the data matrix was stage of disease (early vs. late), which was captured along the first principal component (Fig. 1a: x-axis) and accounted for approximately 36% of total variance. The rate of disease progression was defined by the second principal component (Fig. 1a: y-axis) and accounted for 15% of total variance (Fig. 1b, c and d).

### Functional analysis

**Fig. 2:**
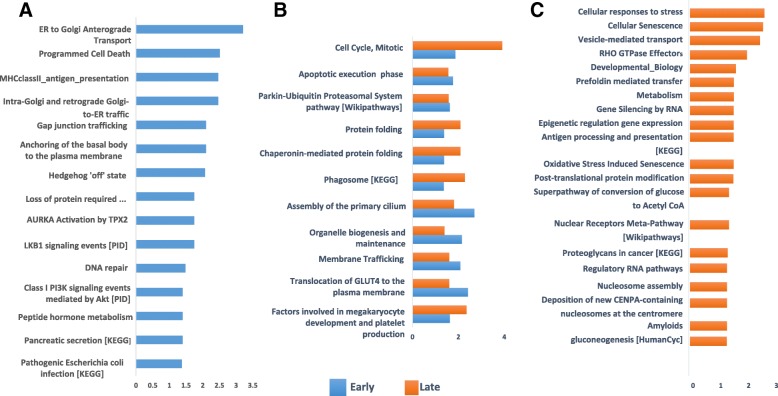
Functional analysis of the proteomic data obtained comparing data from fast versus slow progressing ALS patients. The cross-sectional analysis was based on the list of regulated proteins (FC 1.3, p value < 0.05). Pathways with a p value < 0.05 were considered significantly enriched and–plotted with a -log 10 transformed p value. Functional analysis was performed for Reactome pathways, if not otherwise specified. Only the pathways with the highest enrichment were reported among the selected redundant pathways (mostly cell cycle and mitosis (early) and RHO GTPase (late)). Significantly enriched pathways in the early stage (**a**), late stage (**c**) and in both time points (**b**)

Proteome Sciences’ proprietary tool for functional analysis (FAT) was used to identify significantly enriched pathways and biological processes in the human and mouse proteomics data. A cross-sectional analysis was undertaken at the early (Fig. 2) and late (Additional file 1: Table S1) time points individually using fast vs slow progressing ALS patients as terms of comparison. Fast and slow ALS phenotypic variants were also analysed separately in a longitudinal study, comparing early and late time points (Additional file 1: Table S1, (2–3)). The same analytical approach was used to process data from pre-symptomatic and symptomatic fast and slow progressing SOD1G93A transgenic animal models (Additional file 1: Table S1(2–7): cross-sectional and longitudinal analysis; Additional file 1: Table S4: cross-sectional analysis between SOD1G93A transgenic animals and Wild type littermates).

#### Pathway enrichment

To avoid redundancy, only those pathways with the highest enrichment values were shown when multiple closely related pathways were identified by FAT. The list of selected pathways is presented in descending order of statistical significance (Fig. 2). In the early stage (Fig. 2a), the strongest differential expression between fast and slow progressing ALS patients was related to proteins involved in ER to Golgi anterograde and retrograde transport, programmed cell death, the immune response linked to MHCII antigen presentation and to pathogenic *Escherichia coli* infection. Pathways found to be regulated in both late stage and earlier time point plasma samples included cell cycle (mitosis), apoptosis, protein degradation, post-translational modifications and folding, parkin-ubiquitin proteasome system, organelle maintenance and membrane transport linked pathways (organelle biogenesis and maintenance, phagosome, and translocation of glucose transporter 4 (GLUT4) to the plasma membrane; Fig. 2b). Pathways showing significant enrichment only at the later time point included cellular responses to stress, senescence, vesicular transport, RHO GTPases, RNA regulatory and metabolic processes (Fig. 2c).

**Fig. 3:**
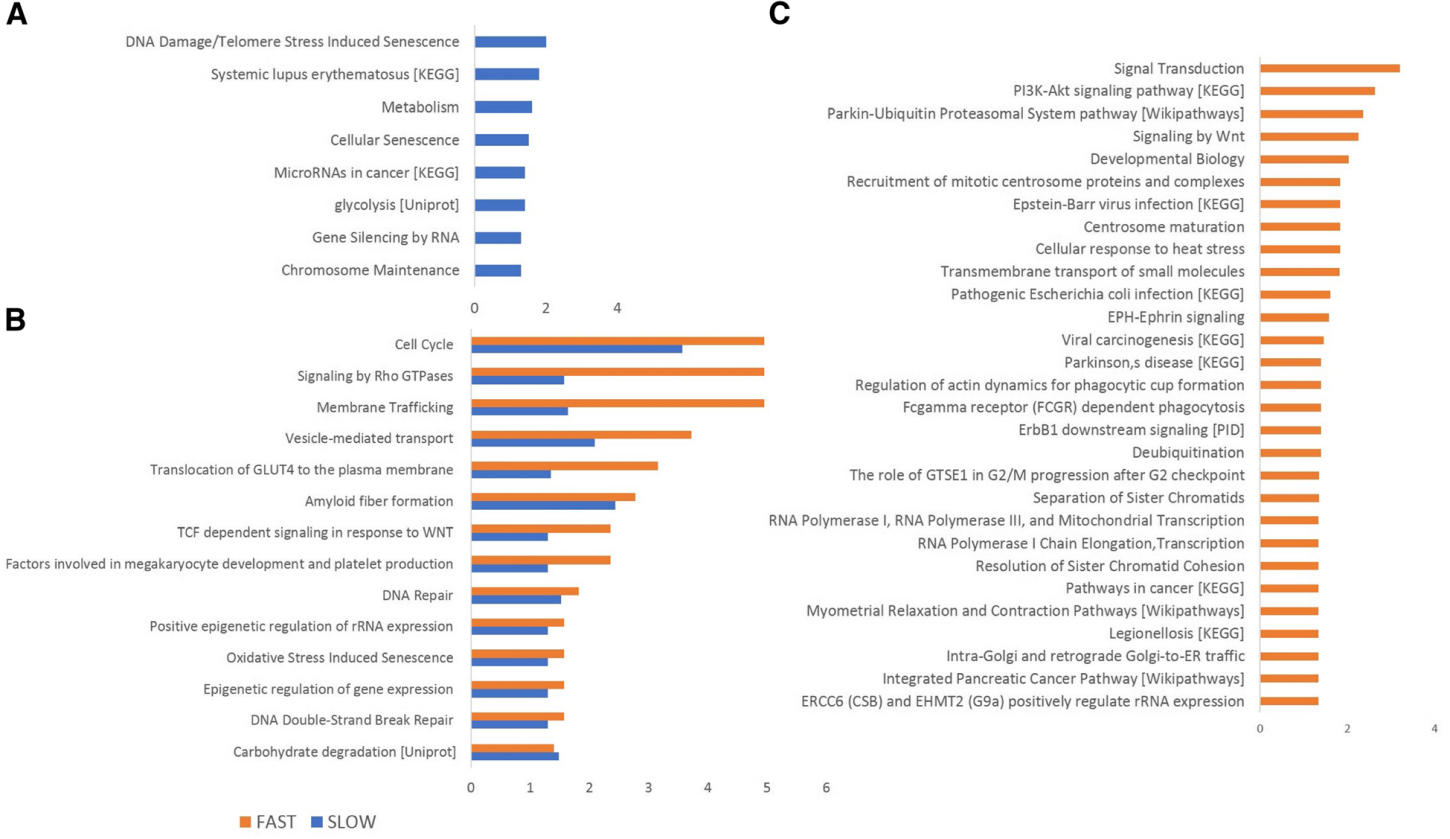
Functional analysis of the proteomic data obtained comparing early versus late disease stage. The longitudinal analysis was undertaken using the list of total regulated proteins (FC 1.3, *p* value < 0.05) in slow and fast progressing ALS individuals independently. Pathways with a p value < 0.05 were considered significantly enriched and plotted with a - log 10 transformed p value in descending order of statistical significance. Functional analysis was performed for Reactome pathways, if not otherwise specified. Only pathways with the highest enrichment were reported among redundant pathways. Significantly enriched pathways in slow progressing patients (**a**), in fast progressing patients (**c**) and shared by slow and fast progressing patients (**b**)

When the slow and fast progressing ALS patients were analysed separately and longitudinally (Fig. 3), the most enriched pathways in slow progressors (Fig. 3a) included DNA damage and telomere stress induced senescence, inflammation and metabolism. In fast progressing ALS patients (Fig. 3c), changes in signal transduction and parkin-ubiquitin proteasome system pathways were among the most enriched, followed by a cluster of five different pathways sharing a role in the immune response, RNA regulation and protein transport. Notably, in common with the cross-sectional study, the longitudinal analysis identified regulation of proteins involved in cell cycle and signalling, Rho GTPase, membrane trafficking, organelle mediated transport and translocation of GLUT4 to the plasma membrane (Fig. 3b).

#### Gene ontology (GO) biological processes

In the cross-sectional study, cytoskeleton organization was found enriched in both early and late time points while phagocytosis, epidermis development, membrane organization, innate immune response, ageing and cell division were among the most enriched biological processes only in the early time point. In the late time point, regulation of endopeptidase activity was the top enriched term along with fibrinolysis and proteolysis (Additional file 1: Figure S3).

In the longitudinal study and in the fast progressing ALS patients, proteins involved in cytoskeletal organization, folding and stabilization, cell adhesion, negative regulation of endopeptidase activity, innate immune response and substantia nigra development were within the most significantly enriched biological features (Additional file 1: Figure S3B). The analysis of the early versus late time points in the slow progressing patients did not show any significantly enriched biological process.

#### Regulated protein candidates

The early disease time point was chosen as the source of most informative differentially expressed plasma protein candidates when comparing ALS-Fast versus ALS-Slow (Table 2; proteins containing two or more peptides shown in a top-down order of fold-changes). Additional file 1, Table 1 shows the most regulated proteins comparing fast versus slow ALS patients in late disease (1), early versus late time points in both fast (2) and Slow (3) progressing ALS patients, as well as those identified in the same cross-sectional (4–5) and longitudinal (6–7) experiment in animal models. The most regulated proteins emerging from the comparison of pre-symptomatic and symptomatic SOD1G93A transgenic mice with their related genetic back-ground WT littermates are reported in Additional file 1: Table S3.

**Table 2:** Regulated proteins in fast compared to slow progressing ALS patients in the early disease stage

	Protein ID	Protein Descriptions	Gene Name	Peptide #	Early _Fast/Early Slow Log Fold Change	adjusted p-value
Up regulated in FAST. Early stage.	P05106	Integrin beta-3	ITGB3	2	2.224407	0.001111
	O95810	Serum deprivation-response protein	SDPR	3	2.102	0.001403
	H7BYX6	Isoform of P13591, Neural cell adhesion molecule 1	NCAM1	2	2.027403	0.000418
	P04003	C4b-binding protein alpha chain	C4BPA	7	2.013732	8.04E-05
	P07437	Tubulin beta chain	TUBB	2	1.908567	0.00790
	P63173	60S ribosomal protein L38	RPL38	2	1.727873	0.00652
	Q14697	Neutral alpha-glucosidase AB	GANAB	2	1.638215	0.00134
	P02649	Apolipoprotein E	APOE	9	1.513701	8.53E-05
	P36957	Dihydrolipoyllysine-residue succinyltransferase component of 2-oxoglutarate dehydrogenase complex, mitochondrial	DLST	2	1.487187	0.00960
	A0A0A0MR02	Isoform of P45880, Voltage-dependent anion-selective channel protein 2	VDAC2	4	1.4397	0.00229
	P05090	Apolipoprotein D	APOD	6	1.293627	0.00240
	P80723	Brain acid soluble protein 1	BASP1	4	1.220442	0.00111
	P15169	Carboxypeptidase N catalytic chain	CPN1	3	1.17725	0.00616
	Q06033–2	Isoform of Q06033, Isoform 2 of Inter-alpha-trypsin inhibitor heavy chain H3	ITIH3	4	1.176924	0.00172
	P48740–2	Isoform of P48740, Isoform 2 of Mannan-binding lectin serine protease 1	MASP1	2	1.126878	0.00134
	P35527	Keratin, type I cytoskeletal 9	KRT9	6	1.061453	0.00827
	Q15582	Transforming growth factor-beta-induced protein ig-h3	TGFBI	3	1.026849	0.00652
	P07355	Annexin A2	ANXA2	6	1.021165	0.00788
Down regulated in FAST Early stage.	P69905	Hemoglobin subunit alpha	HBA1	6	−1.12687	0.00682
	E7ETH0	Isoform of P05156, Complement factor I	CFI	3	−1.14553	0.00190
	O75636–2	Isoform of O75636, Isoform 2 of Ficolin-3	FCN3	4	−1.16238	0.00616
	Q96IY4	Carboxypeptidase B2	CPB2	2	−1.23801	0.00134
	P05452	Tetranectin	CLEC3B	2	−1.32297	0.00173
	P30050	60S ribosomal protein L12	RPL12	2	−1.50426	0.00172
	P20700	Lamin-B1	LMNB1	2	−1.86089	0.00728
	P00505	Aspartate aminotransferase, mitochondrial	GOT2	2	−2.47293	0.00172
	P02042	Hemoglobin subunit delta	HBD	3	−2.49876	0.00040
	Q5T123	Isoform of Q9H299, SH3 domain-binding glutamic acid-rich-like protein 3	SH3BGRL3	2	−2.75577	0.0011

Only proteins identified with at least two unique peptides are shown. Up-regulated proteins are shown in the upper part of the table (grey), with Integrin beta-3 showing the highest fold change. Down-regulated proteins are shown in the bottom part of the table (light blue), with Isoform of Q9H299, SH3 domain-binding glutamic acid-rich-like protein 3, showing the highest fold change of all down-regulated proteins

### Plasma proteomic profile in fast vs slow SOD1 G93A mouse models of ALS and comparability with human pathology

**Fig. 4:**
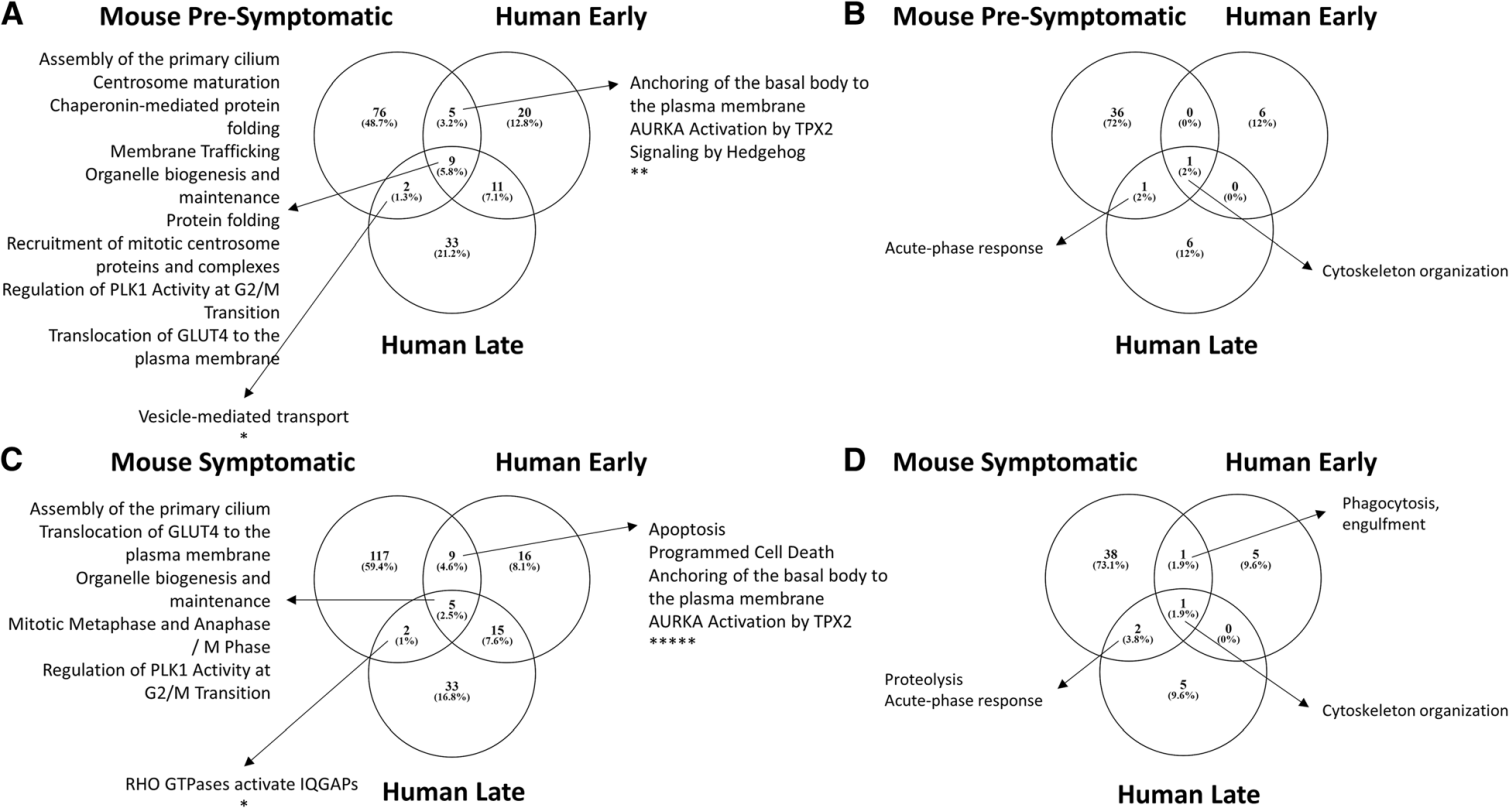
Human-animal model comparison of enriched pathways (**a** and **c**) and biological processes (**b** and **d**) derived from regulated proteins in each specie. **a** Shows the pathways and (**b**) the biological processes that were found enriched in both the mouse model pre-symptomatic stage and in the human ALS proteome at an early and a late disease stage respectively. **c** Shows the pathways and (**d**) the biological processes found enriched in both the mouse model symptomatic stage and the human ALS proteome at an early and late disease stage respectively. Pathways and biological processes, including translocation of GLUT4 to the plasma membrane or acute-phase response, that were found regulated in both mouse model and human plasma proteome at all time points are indicated with an asterisk (*)

Functional analysis for enriched pathways and biological processes (GO terms) in the plasma proteome from the ALS animal models was performed in the same way as for human cases (Additional file 1: Table S2). Enriched features were compared with those seen in the human arm of the study. There was only a partial overlap in enriched pathways between the human and mouse proteome changes comparing data from the different time points in the two species. Chaperonin mediated protein folding and mitotic processes (pre-symptomatic mouse model, early and late human ALS; Fig. 4a); RHO GTPase activators of IQGAPs (symptomatic mouse model, late human ALS) and apoptosis, anchoring of the basal body to the plasma membrane and AURKA Activation by TPX2 (symptomatic mouse model, early human ALS) (Fig. 4c) were among the pathways shared by human and mouse ALS.

However, four pathways; assembly of the primary cilium, organelle biogenesis and maintenance, regulation of PLK1 Activity at G2/M transition and translocation of GLUT4 to the plasma membrane were found enriched in both animal models and human disease at all stages considered (Fig. 4a, c).

With regards to biological processes, cytoskeleton organization was the only feature enriched when comparing fast versus slow plasma proteomes from animal and human at the early and late time points (Fig. 4b, d). The acute-phase response GO term was consistently enriched in the pre-symptomatic and symptomatic mouse proteome, but only enriched in the late stage of human disease. The dysregulation of proteolysis pathway was observed only at the late stage in both species. Phagocytosis engulfment was shared between the human early and the symptomatic mouse time points (Fig. 4d).

### Immunodetection studies

Using immunodetection, we have re-tested the plasma expression of protein candidates belonging to the regulated pathways identified by plasma TMT proteomic analysis in ALS patients and in SOD1G93A transgenic mice, including metabolic processes (APOE, APOA1), acute response, inflammation and cell senescence (ITGB3, Galectin-3, TGFB1). These proteins appear also as regulated in the proteomic analysis comparing WT and SOD1G93A transgenic mice in both genetic back-grounds (Additional file 1: Table S5). The re-test experiment was performed using plasma samples from the ALS patients of the discovery cohort (*n* = 12), ALS from a re-test cohort (*n* = 47) and from healthy controls (*n* = 29; Table 1) as well as plasma samples from WT129Sv, Pre-SOD1129Sv, Sym-SOD1129Sv, WTC57, Pre-SOD1C57 and Sym-SOD1C57 mice (*n* = 36, 6 per sub-group).

**Fig. 5:**
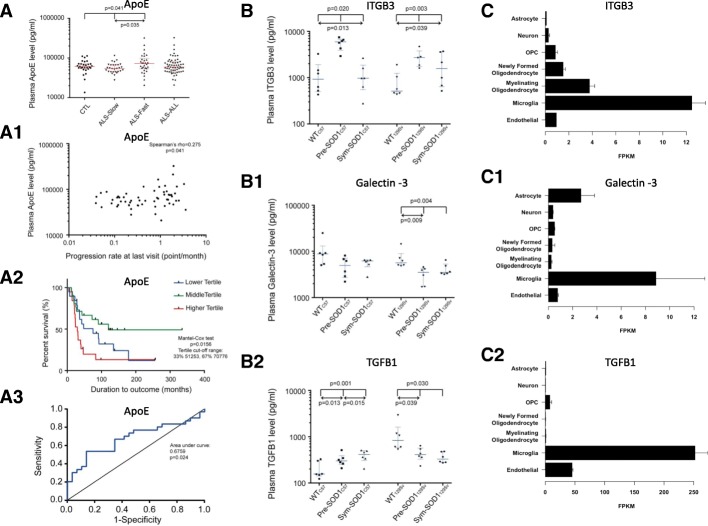
Re-test of protein candidates using immunoassays. ELISA and Meso Scale Discovery (MSD) analysis of selected protein candidates in plasma samples. **a.** APOE is up-regulated in plasma samples from ALS-Fast compared to ALS-Slow; **a1**: positive correlation between APOE plasma levels and PRL in ALS patients; **a2**: reduced survival for ALS patient with higher APOE levels (above 70,776 pg/ml). **a3**: APOE levels separate ALS-Fast from ALS-Slow **b.** ITGB3 is up-regulated in plasma from pre-symptomatic transgenic SOD1G93 animal models (Pre-SOD1129Sv, Pre-SOD1C57) compared to related WT animals, while ITGB3 plasma expression in the respective symptomatic animals are significantly reduces to WT levels. **b1** Galectin-3 is significantly downregulated in pre-symptomatic (Pre-SOD1129Sv) and symptomatic in fast progressing SOD1G93A transgenic mice (Sym-SOD1129Sv) compared to 129Sv WT animals (WT129Sv). **b2** TGFB1 is significantly upregulated in plasma from Pre-SOD1C57 and from Sym-SOD1C57 compared to WTC57, while it down-regulated in Pre-SOD1129Sv and Sym-SOD1129Sv compared to WT129Sv. In each figure, reported upper p-values relate to Kruskall-Wallis while lower *p*-values to Dunn’s multiple comparisons tests. **c** data mining using a RNA-Seq transcriptome and splicing database of glia, neurons, and vascular cells of the cerebral cortex. This interactive splicing browser shows predominant microglia expression of ITGB3 (**c**), Galectin-3 (**c1**) and (**c2**) TGFB1 (http://www.brainrnaseq.org/) in a range of cortical cells [28]. PRL: progression rate to last visit. WT: wild type. OPC: oligodendrocyte precursor cells. FPKM: fragments per kilobase of transcript sequence per million mapped fragments

*ALS patients*: APOE was up-regulated in plasma from ALS-Fast compared to ALS-Slow and healthy controls (*p* = 0.035 and *p* = 0.041 respectively; Fig. 5a). Regression analysis showed a modest correlation between APOE plasma levels and PRL (p = 0.041; Fig. 5a1). Kaplan Mayer analysis identified reduced survival in the higher APOE tertile compared to middle and lower tertiles (*p* = 0.0156; Fig. 5a2) while evaluation of diagnostic performance by receiver operating characteristic (ROC) analysis showed that APOE levels separated ALS-Fast from ALS-Slow (Area: 0.6759; Std. Error = 0.07162; *p* = 0.0204; Fig. 5a3).

APOE analysis in plasma from the discovery cohort (n = 12) showed a trend of over-expression in ALS-fast compared to ALS slow which did not reach significance (data not shown; *p* = 0.069). There was no statistically significant regulation of Galectine-3, TGFb1, ApoA1 and ITGB3 plasma levels at group analysis comparing healthy controls to ALS-FAST and ALS-Slow in both discovery and re-test cohorts, although a trend of up-regulation in ALS-Fast compared to ALS-Slow was seen in both cohorts for these protein candidates (data not shown).

ITGB3 and Galectin-3 WB analysis was performed on plasma samples from the discovery and re-test cohort, including ALS-Fast (n = 12), ALS-Slow (n = 2) and healthy controls (*n* = 10). WB showed a trend of up-regulation for both Galectin-3 and ITGB3 in ALS-Fast compared to ALS-slow which was not significant (data not shown). These results are in line but not in complete agreement with the level of differential regulation seen in the proteomic experiment of the early and late time points (ITGB3 adjusted *p* value = 0.001111; Galectin adjusted p value = 0.002369; Table 2, Additional file 1: Table S1).

SOD1*G93A transgenic mice*: ITGB3 was significantly up-regulated in plasma from pre-symptomatic SOD1G93A transgenic mice of both fast (129S) and slow (C57) genetic backgrounds compared to both WT and symptomatic mice (Fig. 5b; Pre-SOD1129Sv *p* = 0.003, Pre-SOD157 p = 0.020). Within the same genotypes, ITGB3 expression decreased significantly in symptomatic SOD1G93A transgenic mice compared to pre-symptomatic (Sym-SOD1C57 *p* = 0.013; Sym-SOD1129S *p* = 0.039; Fig. 5b). Galectin-3 was down-regulated in plasma from pre-symptomatic and symptomatic SOD1G93A transgenic mice with fast genetic background compared to their WT littermates (Pre-SOD1129Sv *p* = 0.009; Sym-SOD1129Sv *p* = 0.004; Fig. 5b1), but not in the slow progressing genetic background (Fig. 5b1). TGFB1 plasma expression increased significantly in pre-symptomatic and symptomatic slow SOD1G93A transgenic mice compared to their WT littermates (Fig. 5b2: Pre-SOD157 *p* = 0.013; Sym-SOD1C57 *p* = 0.001). Conversely, TGFB1 was down-regulated in pre-symptomatic and symptomatic fast SOD1G93A transgenic mice compared to their WT littermates (Pre-SOD1129Sv p = 0.039; Sym-SOD1129S p = 0.039). There was no statistically significant change of APOE and APOA1 plasma expression between WT and SOD1G93A transgenic mice of both genetic backgrounds (data not shown). For all 5 protein candidates, immunodetection supported the same pattern of expression found in proteomics.

Analysis of RNA expression of Galectin-3, TGFB1 and ITGB3 in a range of cortical cells [28] showed predominant microglia expression for all three proteins (Fig. 5c, c1, c2 - http://www.brainrnaseq.org/).

## Discussion

This study has three unique aspects: 1) a novel quantitative proteomics approach which combines tissue and fluid in a single experiment using TMT® labelling and liquid chromatography combined to mass spectrometry, 2) the use of PBMC, peripheral reporters of central neuro-inflammation [29] as source tissue for plasma biomarkers identification and 3) the inclusion of two species (human and mouse) where ALS is linked to different disease causative factors: multiple copies of mutated SOD1 transgenes in the animal and not yet fully characterized genetic and most probably environmental factors in human (none of the patients carried one of the genetic mutations so far identified in ALS).

We have observed that the rate of ALS progression, and above all the disease stage, are linked to substantial changes in the abundance of a wide range of plasma proteins. In ALS patients and animal models, regulated plasma proteins support a range of inflammatory events and metabolic modifications, while senescence-related alterations are mostly seen with the disease progression in the human ALS proteome. Studying similar phenotypic variants in mouse models and patients with ALS, we have identified only a partial overlap in the overall plasma proteomes acquired in time points which may not necessarily be comparable in the natural history of the disease in humans and rodents. Furthermore, the monogenetic driver of disease in an inbred animal model may only partially reflect the pathological processes of adaptation seen in sporadic human disease, explaining this discrepancy. It is nevertheless encouraging that we could dissect common features that could be developed as translational biomarkers to bridge pre-clinical and human studies. These shared biological features appear to be independent from those genetic and environmental determinants driving the disease in humans and animals, representing an ALS-specific signature and priority targets for any investigation into biomarkers and therapeutics in both pre-clinical and clinical setting.

The cross-sectional proteomic studies in human and animals were based on a relatively small sample size. The risk of obtaining non-specific biological signals was mitigated by the inclusion of phenotypic variants and disease time points to test the longitudinal profile of the disease, while a re-test experiment in larger cohorts of WT and transgenic animals as well as of ALS patients and healthy controls, helped to confirm the regulation of relevant protein candidates observed using proteomics, particularly in the mouse model. Further investigation of the regulated immuno-metabolic and senescence pathways showed how plasma expression of APOE differentiates ALS patients based on rate of disease progression, while the study of other biomarkers like ITGB3, Galectin-3 and TGFB1 confirmed the pattern of regulation across WT and SOD1G93A transgenic animal models seen in the proteomic experiment. Evidence of Apolipoprotein E genotype being a determinant of age at onset in ALS patients and in particular, of the ε4 allele being a determinant of survival and a modifier of clinical expression particularly for the risk of bulbar-onset ALS in men has been reported and discussed in the literature [30] . From a biomarker perspective, APOE plasma levels have also been correlated with both rate of deterioration and survival [31].

The re-test study by immunodetection in ALS patients did not show the same degree of regulation for ITG3, Galectin-3 and TGFB1 shown in ALS-Fast compared to ALS-Slow using deep proteomics. This can be explained by the small sample size of the discovery cohort used in the proteomic experiment and by the concomitant high phenotypic (and genetic) variability of the disease in human. MS and antibody-based detection techniques have also different analytical sensitivity and specificity, which could also depend on the pre-analytical albumin depletion utilized in proteomics but not in ELISA, as albumin’s chaperoning and protein-binding activity may affect the abundance of specific proteins [32]. In the more homogeneous animal models of ALS, proteomic and immunodetection provided comparable results.

### The plasma/PBMC proteome as a reporter of disease progression in human ALS

PCA indicates that differences in the plasma proteome are more significant between early and late stages of disease, independent of speed of progression (Fig. 1). The selection of ALS patients in an “early” disease stage is complicated by unavoidable diagnostic delays and by poor detection of early signs of “phenoconversion” in asymptomatic individuals. Therefore, in our study, participants were enrolled and sampled at or shortly after diagnosis, and re-sampled after an interval of 6 to 24 and of 48 to 56 months for fast and slow progressing ALS patients respectively (fast and slow ALS patients had comparable ALSFRS-R at the late stage). The change of the plasma proteomic profile in this time-frame includes the early activation of the innate immune response (MCH II signalling), initiation of apoptosis and dysfunction of the proteasome systems which only partially overlap with the pre-symptomatic mouse plasma proteome, while later alterations related to metabolism and glucogenesis as well as RHO GTPase activation are in common with the animal model proteome (Fig. 2). In our study, RHO GTPase activity is closely linked to a marked regulation of the cytoskeletal proteins organization in both species. RHO GTPase binding to plasma membrane-associated actin cytoskeleton is fundamental to maintain cell-cell and/or cell extracellular matrix (ECM) adhesion [33]. The early immune response activation in our plasma proteome endorses previous reports of a systemic inflammatory response in ALS, involving monocyte/macrophages and a decrease of peripheral T-regulatory cells (Treg) particularly in fast progressing ALS patients, but may also relate to those auto-immune disorders which are co-morbid or precede ALS as previously reported [5, 17, 34–36]. However, none of the ALS cases in study had such a history and their blood levels of acute phase reactants, including CRP and ferritin, were within normal limits.

The prominent expression of proteins involved in the translocation of GLUT4 to the plasma membrane observed in all disease stages under investigation in human and mice, suggests an altered glucose uptake from the blood-stream and a dysmetabolic state (Fig. 2). Lactate, a by-product of cell metabolism which has been reported as raised in blood and CSF from ALS patients, is critically involved in glucose utilization for energy production [37–39]. Recently, it has been proposed that the adenosine triphosphate (ATP)-dependent lactate flow between muscle and neurons at the neuromuscular junction (NMJ) may become disrupted in ALS [40]. Extracellular lactate has been shown to cause T cell entrapment at sites of inflammation by inhibition of CD4+ and CD8+ T cells motility, inducing chronic inflammation and a switch of CD4+ T helper cells to a Th17 phenotype with the release of pro-inflammatory cytokines [41]. Hence lactate may facilitate chronic inflammation, a key feature of neurodegeneration, while causing NMJ disruption and motor neuron death. Lactate dyscrasia and translocation of GLUT4 to plasma membrane have also been reported to interfere with muscle contraction by loss of GTPase activity [42–46]. The altered GLUT4 translocation and lactate dyscrasia are among those features potentially bridging human to animal pathology, representing a biologically plausible biomarker and treatment target in ALS.

### Inflammation, cell senescence and microglial biomarkers in ALS across species

When the differential plasma proteomic profiles of pre-symptomatic and symptomatic transgenic animals were compared to early and late ALS cases, we found inter-species commonalities which included, among others, changes in the acute phase and innate immune responses, protein folding and GTPase Rho signalling (Fig. 4). Further analysis of differentially regulated features in our study pointed to microglia activation: 1) the early up-regulation of proteins involved in apoptosis, phagocytosis and of the proteasome system reflecting microglia/macrophage activation which leads to removal of cellular debris and protein aggregates by phagocytosis [47] and 2) ITGB3, Galctin-3 and TGFB1 are selective brain microglia markers, according to a large RNA-Seq transcriptome and splicing database of glia, neurons, and vascular cells of the cerebral cortex (http://www.brainrnaseq.org/; Fig. 5, C,C1,C2).

Integrins, Galectin-3 and TGFB1 have been implicated in different biological processes including angiogenesis, fibrosis and wound healing [48, 49]. These proteins are also central to the development of the acute phase response leading to chronic tissue injury [50], a molecular feature shared by animal and human ALS plasma proteomes. Critically, ITGB3 has been shown to be a key factor in cell senescence and in the interplay between membrane and ECM through a molecular cascade that includes activation of TGFB1 [51]. Our study also show activation of telomere stress induced senescence with disease progression, implicating ITGB3 and TGB1 further in replicative senescence, where telomere length becomes fundamental in loss of cellular homeostasis observed with aging [52]. Astrocyte over-expression of TGFB1accelerates disease progression in ALS mice [53]. ALS skeletal muscle and plasma also show enhanced TGFB1signaling, which can lead to muscle fibrosis [54, 55]. The up-regulation of ITGB3 and TGFB1adds to our previous finding of increased levels of pro-inflammatory cytokines like IL-6 in plasma from ALS patients, a concerted response described as senescence-associated secretory phenotype [6, 56–58]. These microglial targets are ALS-specific as they seem relevant to both mouse and human, where the disease is likely to follow different pathological routes to arrive to the same end-point: the loss of motor neurons.

### ALS: Choosing the right therapeutic target and timing for intervention

In humans, the lack of any means of accurate prognostication makes ALS clinical heterogeneity the main obstacle to the development of effective treatments [34]. Conversely, in the relatively homogeneous SOD1 animal models of ALS, phenotypic variations depend only on expression levels of mutant SOD1, gender, genetic background and possibly on breeding conditions, all features that can be manipulated in a controlled experimental setting [59]. We therefore believe that any novel treatment strategy for ALS will have to be tested in a more personalized approach, using biomarkers and genetics for clinical stratification [60]. If pre-clinical work is contemplated like, for example, testing drug efficacy in a mouse model, than it may be reasonable to choose the molecular target of drug engagement among those shared by the model and human pathology, so that translation to ALS patients may have more chances to succeed. This study offers insight into human-rodent molecular pathways that could be at the center of future treatment strategies for ALS.

Our study has shown a convergence between species in molecular mechanisms which support the regulation of the immune response and of cell metabolism. These shared regulated pathways support the potential for early immune-modulatory strategies, including the use of anti-inflammatory compounds, stem-cell based immune-effective treatment or protein kinase inhibitors, used to stem microglia-mediated neuroinflammation [21, 61, 62]. While fighting this innate immune response as early as possible may be plausible, it is worth considering that macrophages at this stage and potentially throughout the disease course clear debris from cells undergoing degeneration. This “beneficial” inflammatory response may later shift towards a chronic and harmful process [6, 47, 63–67]. Therefore, timing of immunomodulation in ALS is crucial and interventions must aim at the window of therapeutic opportunity to arrest any development to chronic inflammation, which is an integral part of cell senescence and of the SASP response. Inducers of heat shock response have been shown to efficiently inhibit and reduce chronic inflammation in obesity and the same therapeutic paradigm may be relevant in neuroinflammation [68].

Our findings support also treatment strategies which target the enzymatic chain of glucose metabolism leading to the production of lactate, based on the combination of drugs that inhibit lactate accumulation at the NMJ and its effect in reducing T-cell migratory capabilities, enhancing respiratory chain function, and/or promoting re-innervation [40]. Interestingly, nutritional supplements acting at the interface between inflammation and metabolism have been shown to dampen the inflammatory environment. For example, omega-3 essential fatty acids decrease the levels of IL-1, IL-6, TNFα and CRP [69] and improve cognitive function in aged mice [70].

## Conclusions

This study provides one of the most in-depth qualitative and quantitative proteomics studies performed in ALS, using the plasma/PBMC interface to investigate crucial aspects of phenotypic heterogeneity of this condition and across species. Researchers working on ALS and on other neurodegenerative disorders will be able to draw conclusions from the data we have generated to steer the development of novel biomarkers and therapeutic strategies in ALS.

## Additional files

Additional file 1: **Figure S1.** represents and scheme of the workflow followed to perform this study. **Figure S2.** shows the PCA before batch effect correction, **Figure S3.** shows enriched biological processes for the cross-sectional and longitudinal study. **Table S1.** shows the top regulated proteins in plasma from 1) fast versus slow progressing ALS patients at the late stage disease, early versus late time points for slow and fast progressing ALS patients and mouse model: cross sectional and longitudinal studies. **Table S2.** where the functional analysis of the animal model proteomic data are presented. **Table S3.** Presents the top regulated proteins in plasma from Wild type versus SOD1G93A transgenic mice at the pre-symptomatic and symptomatic stage of disease, for both genetic backgrounds under investigation. **Table S4.** contains the results for the functional analysis for the proteomic study comparing wild type versus transgenic ALS SOD1G93A mice. **Table S5.** Shows the proteomic data of the immunosenescence protein candidates selected for the re-test experiments. (DOCX 295 kb). 

Additional file 2: Detailed protocols for Sample fractionation, LC-MS/MS analysis and peptide identification and quantification: methods are explained in more detail in this additional file. (PDF 14 kb)

Additional file 3: contains all the raw data generated in the human analysis. (CSV 495 kb)

Additional file 4: contains all the raw data generated in the animal model analysis. (CSV 3617 kb)

## Abbreviations

ALS: Amyotrophic lateral sclerosis
ALSFRS-R: Amyotrophic lateral sclerosis Functional Rating Scale-Revised
CRP: C-reactive protein
ECM: Extracellular matrix
EDTA: Ethylenediaminetetraacetic acid
ER: Endoplasmatic Reticulum
FBS: Fetal bovine serum
FTD: Frontotemporal dementia
HRP: Horseradish peroxidase
IL: Interleukin
ITGB3: Integrin Beta 3
LC-MS/MS: Liquid chromatography tandem mass spectrometry
LIMMA: Linear models for microarray data
logFC: Log2 fold changes
MHC: Major histocompatibility complex
NMJ: Neuromuscular junction
PBMC: Peripheral Blood Mononuclear Cells
PBS: Phosphate buffered saline
PCA: Principal component analysis
PRL: Disease progression to last visit
PSM: Peptide spectrum matches
RT: Room temperature
SASP: Senescence-associated secretory phenotype
SCX: Strong Cation exchange
SOD1: Superoxide dismutase 1
TBST: Tris buffered saline- tween
TCEP: Tris(2-carboxyethyl) phosphine 10
TDP-43: TAR DNA-binding protein 43
TEAB: Triethylammonium bicarbonate
TGF-β1: Transforming growth factor beta 1
TMT: Tandem mass tag
TNFα: Tumor necrosis factor alpha
