# Supplementary material for: Tissue-enhanced plasma proteomic analysis for disease stratification in amyotrophic lateral sclerosis
Source: Mol Neurodegener. 2018 Nov 7;13:60. doi: 10.1186/s13024-018-0292-2 (PMC6223075; doi:10.1186/s13024-018-0292-2)
Supplement: Supplementary file 1 — Figure S1. represents and scheme of the workflow followed to perform this study. Figure S2. shows the PCA before batch effect correction, Figure S3. shows enriched biological processes for the cross-sectional and longitudinal study. Table S1. shows the top regulated proteins in plasma from 1) fast versus slow progressing ALS patients at the late stage disease, early versus late time points for slow and fast progressing ALS patients and mouse model: cross sectional and longitudinal studies. Table S2. where the functional analysis of the animal model proteomic data are presented. Table S3. Presents the top regulated proteins in plasma from Wild type versus SOD1G93A transgenic mice at the pre-symptomatic and symptomatic stage of disease, for both genetic backgrounds under investigation. Table S4. contains the results for the functional analysis for the proteomic study comparing wild type versus transgenic ALS SOD1G93A mice. Table S5. Shows the proteomic data of the immunosenescence protein candidates selected for the re-test experiments. (DOCX 295 kb). [file 13024_2018_292_MOESM1_ESM.docx]

# Additional file 2. Supplementary figures and tables


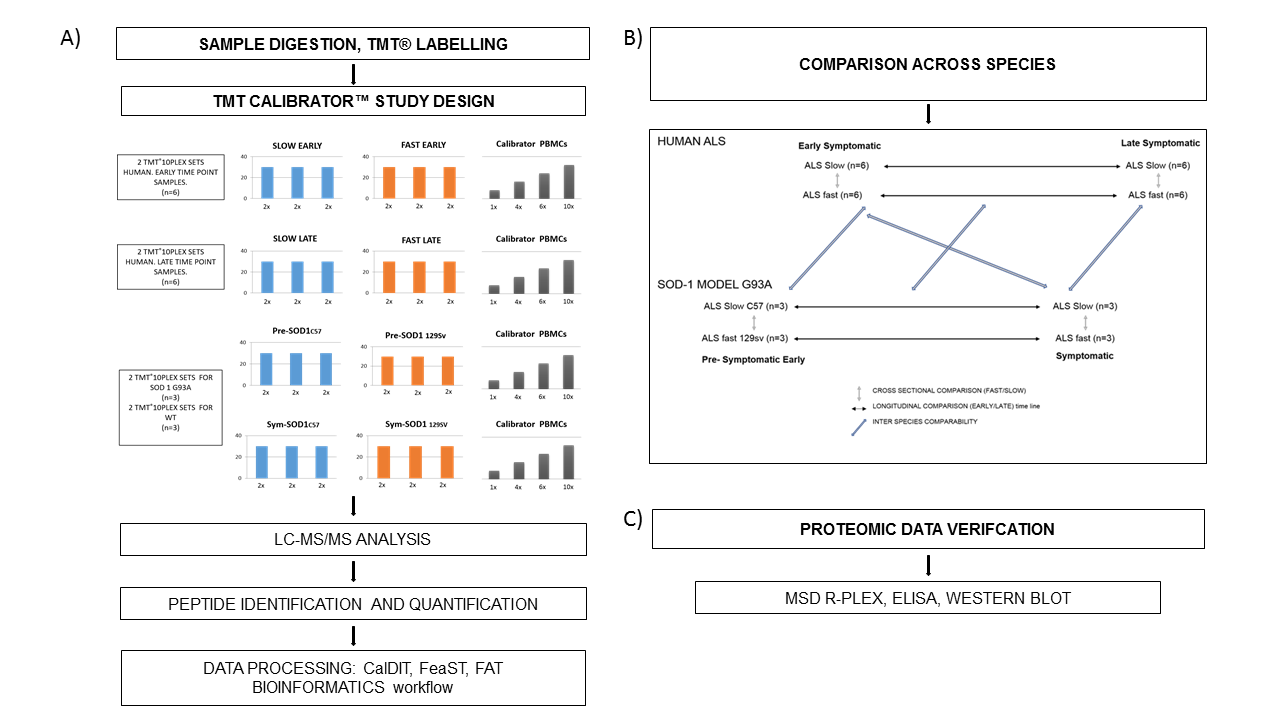


**Supplementary figure 1: Experimental workflow**

TMT calibrator experimental design. **A**. Following digestion, samples were divided in different TMT calibrator Sets. For the human study, plasma samples were divided into four TMT^®^10plex sets, each set containing three fast and three slow progressing ALS patients (ALS-Fast, ALS-Slow) plasma digests combined with the four-point of human PBMC calibrator samples. Two of these sets contained the early time point samples (n=6) and the remaining two sets contained the late time point samples (n=6). The same experimental design was followed in the animal model to compare fast vs slow transgenic at two different time points and Wild type mice. Four different TMT^®^10plex sets were used to analyse 24 samples (n=3 for each condition, genetic background and time point). TMT^®^10plexes included 1) pre-symptomatic and symptomatic time point in the fast genetic back-ground (Pre-SOD1129Sv, Sym-SOD1129Sv), 2) pre-symptomatic and symptomatic time points from the slow genetic background (Pre-SOD1C57 and Sym-SOD1C57) and 3) two 10plex to compare the corresponding 129Sv and C57 wild type animals. The four TMT ^®^10plex sets used for the mouse model analysis, contained the same four-point PBMC calibrator curve formed by a pool of PBMC from all 24 animals under study. All mass spectrometry files were inspected independently and passed internal quality control metrics. Outputs of computational proteomics were assembled into a single dataset and processed by Proteome Sciences’ proprietary workflows for TMTcalibrator^TM^, including data integration (CalDIT), pre-processing and feature selection (FeaST). **B.** Top regulated proteins and significantly enriched pathways as well as biological processes were compared across species as shown in the diagram. **C.** Protein candidates found in regulated pathways / biological features and relevant to both species ALS proteome were re-tested using orthogonal techniques.


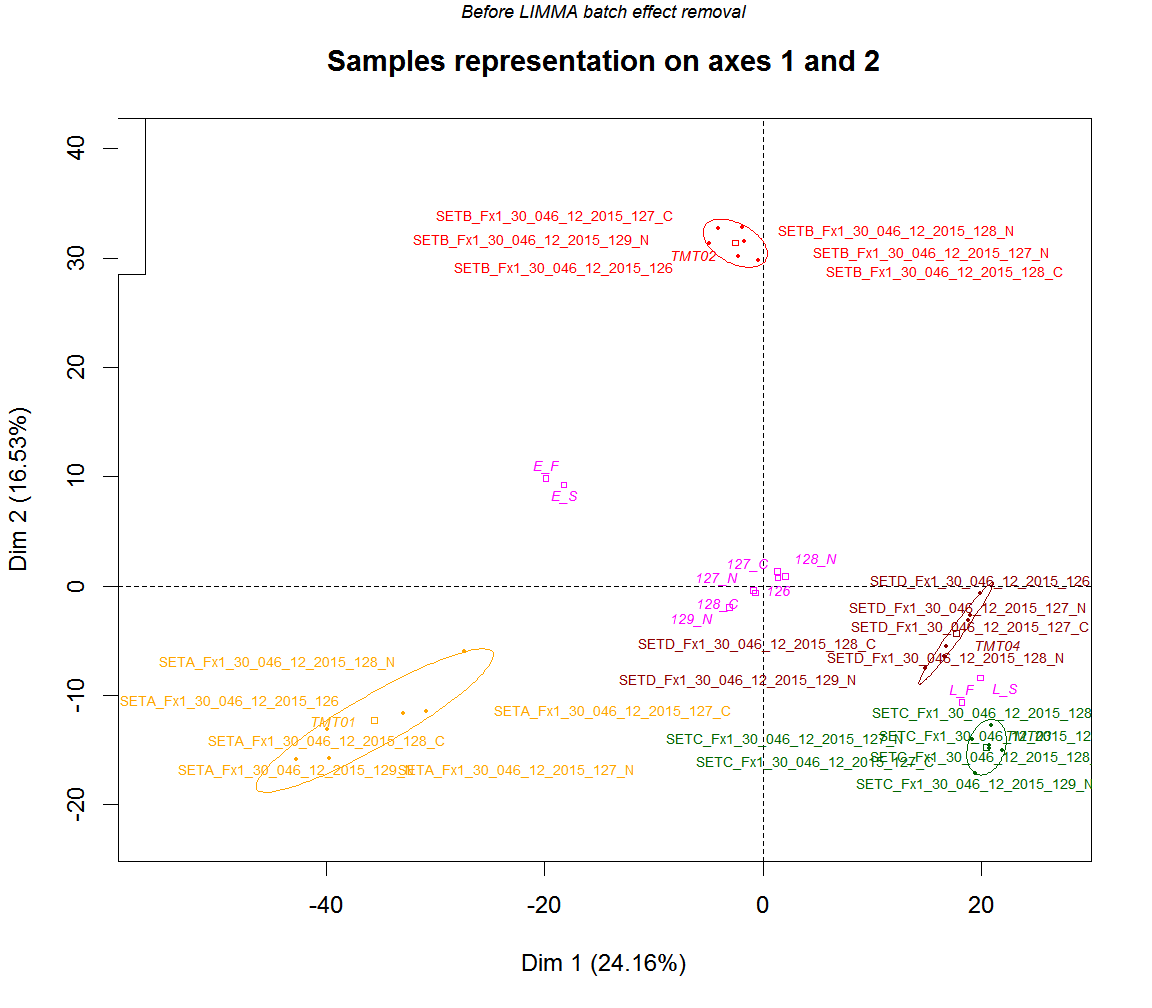


**Supplementary figure 2: PCA before LIMMA Batch effect removal.**

The batch effect created by multiple TMT^®^10 plexes, can be appreciated in this image. A LIMMA-based batch effect correction procedure was applied using a linear model constructed on the TMT^®^ 10plex batch number and TMT^®^ channel and specifying the experimental groups to remove this effect.


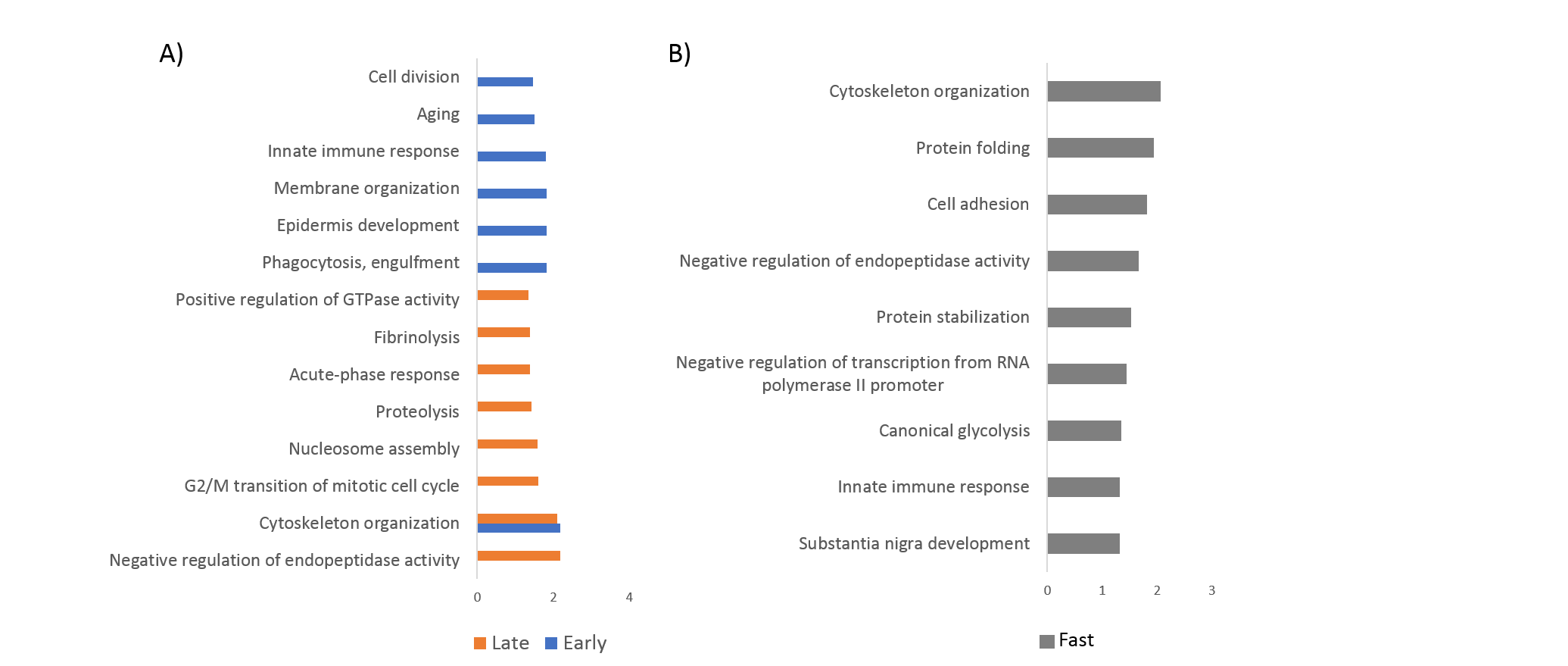


**Supplementary Figure 3. Biological functions and enriched GO terms.**

GO-biological process terms with a p value < 0.05 were considered significantly enriched (significance of enrichment expressed as –log 10 p value). In the cross-sectional study (A), cytoskeleton organization was the only biological process found significantly enriched in both early and late stage disease, while phagocytosis/engulfment was the top enriched biological process in the early time point and negative regulation of endopeptidase activity showed the highest significance in the late stage of the disease. For the longitudinal study and for the fast progressing ALS patients (B), cytoskeleton organization was also the most enriched GO term when comparing early and late time points in the longitudinal study.

**Supplementary Table 1.** Top regulated proteins in plasma from fast versus slow progressing ALS patients at the late stage disease (cross-sectional study (1), early versus late time points for slow and fast progressing ALS patients (ALS-Slow, ALS-Fast), longitudinal study (2) and (3). For the mouse model, cross sectional comparisons between fast and slow at a pre-symptomatic time point (4) and at the symptomatic time point (5). Longitudinal studies in the mouse model (pre-symptomatic vs symptomatic) for the fast (6) and slow progressing (7) mouse models.

| HUMAN STUDY | | | | | | |
| --- | --- | --- | --- | --- | --- | --- |
| 1. CROSS SECTIONAL STUDY (Fast vs Slow), late stage, top 20 regulated proteins in plasma | | | | | | |
| **REGULATION** | **Protein ID** | **Protein Descriptions** | **Gene Name** | **Peptide number** | **Log 2 FC_late _Fast/late Slow** | **adjusted P.Value** |
| Up-regulated in ALS-Fast: late time point, top ten proteins identified by 2 or more peptides. | P62158 | Calmodulin | CALM1 | 2 | 2.739039239 | 0.003812 |
|  | O00151 | PDZ and LIM domain protein 1 | PDLIM1 | 3 | 2.376079261 | 0.000553 |
|  | P08133-2 | Isoform of P08133, Isoform 2 of Annexin A6 | ANXA6 | 2 | 2.119069596 | 0.002278 |
|  | F8W1R7 | Isoform of P60660, Myosin light polypeptide 6 | MYL6 | 4 | 1.822412264 | 0.008304 |
|  | P52907 | F-actin-capping protein subunit alpha-1 | CAPZA1 | 2 | 1.751028461 | 0.000361 |
|  | P21333-2 | Isoform of P21333, Isoform 2 of Filamin-A | FLNA | 35 | 1.717104998 | 0.002278 |
|  | P05106 | Integrin beta-3 | ITGB3 | 2 | 1.704090775 | 0.003803 |
|  | Q9H1E3-2 | Isoform of Q9H1E3, Isoform 2 of Nuclear ubiquitous casein and cyclin-dependent kinase substrate 1 | NUCKS1 | 2 | 1.686292334 | 0.001022 |
|  | P04114 | Apolipoprotein B-100 | APOB | 3 | 1.591983642 | 0.005734 |
|  | P07996 | Thrombospondin-1 | THBS1 | 6 | 1.545267608 | 0.007142 |
| Down-regulated ALS-Fast: late time point, top ten proteins identified by 2 or more peptides. | P68871 | Hemoglobin subunit beta | HBB | 8 | -1.283300481 | 0.009746 |
|  | P32119 | Peroxiredoxin-2 | PRDX2 | 6 | -1.493420521 | 0.000681 |
|  | P23246 | Splicing factor, proline- and glutamine-rich | SFPQ | 5 | -1.571352237 | 0.000481 |
|  | P07339 | Cathepsin D | CTSD | 2 | -1.588720577 | 0.001889 |
|  | Q08380 | Galectin-3-binding protein | LGALS3BP | 3 | -1.611800131 | 0.002369 |
|  | P30050 | 60S ribosomal protein L12 | RPL12 | 2 | -1.636886293 | 0.000553 |
|  | P62805 | Histone H4 | HIST1H4A | 5 | -1.640627509 | 0.000333 |
|  | P02042 | Hemoglobin subunit delta | HBD | 3 | -1.717799008 | 0.003197 |
|  | P62701 | 40S ribosomal protein S4, X isoform | RPS4X | 2 | -1.753180332 | 0.000642 |
|  | O00479 | High mobility group nucleosome-binding domain-containing protein 4 | HMGN4 | 2 | -2.214655303 | 0.000302 |
| 2) LONGITUDINAL STUDY (Early vs late time points): ALS-Fast | | | | | | |
| **REGULATION** | **Protein ID** | **Protein Descriptions** | **Gene Name** | **Peptide number** | **Log 2 FC_early _Fast/Late Fast** | **adjusted P.Value** |
| Up-regulated in the late time point for ALS-Fast, top ten proteins identified by 2 or more peptides. | P15169 | Carboxypeptidase N catalytic chain | CPN1 | 3 | 3.807807428 | 1.93E-08 |
|  | P02656 | Apolipoprotein C-III | APOC3 | 3 | 3.63301436 | 2.53E-07 |
|  | P10643 | Complement component C7 | C7 | 4 | 2.514217497 | 2.01E-08 |
|  | Q15582 | Transforming growth factor-beta-induced protein ig-h3 | TGFBI | 3 | 2.437278722 | 1.51E-06 |
|  | P62937 | Peptidyl-prolyl cis-trans isomerase A | PPIA | 4 | 2.374550326 | 0.000507 |
|  | F5GY80 | Isoform of P07358, Complement component C8 beta chain | C8B | 2 | 2.035866873 | 0.000152 |
|  | Q96IY4 | Carboxypeptidase B2 | CPB2 | 2 | 1.965632846 | 1.24E-05 |
|  |  | Ig mu heavy chain disease protein | Ig mu heavy chain disease protein OS=Homo sapiens | 5 | 1.913768001 | 7.57E-06 |
|  | Q16555-2 | Isoform of Q16555, Isoform 2 of Dihydropyrimidinase-related protein 2 | DPYSL2 | 4 | 1.910543572 | 0.000152 |
|  | P01023 | Alpha-2-macroglobulin | A2M | 44 | 1.900859528 | 5.19E-06 |
| Down regulated in the late time point for fast progressing patients. Top ten with 2 or more peptides. | P67936 | Tropomyosin alpha-4 chain | TPM4 | 5 | -2.183582874 | 0.018685 |
|  | E5RK69;P08133-2 | Isoform of P08133, Isoform 2 of Annexin A6 | ANXA6 | 2 | -2.224366147 | 0.003341 |
|  | P30153 | Serine/threonine-protein phosphatase 2A 65 kDa regulatory subunit A alpha isoform | PPP2R1A | 2 | -2.265585231 | 9.87E-05 |
|  | P08514-2 | Isoform of P08514, Isoform 2 of Integrin alpha-IIb | ITGA2B | 2 | -2.537194962 | 0.000456 |
|  | Q92841-1 | Isoform of Q92841, Isoform 2 of Probable ATP-dependent RNA helicase DDX17 | DDX17 | 2 | -2.681055124 | 0.000172 |
|  | P62158 | Calmodulin | CALM1 | 2 | -3.028962141 | 0.004223 |
|  | P00505 | Aspartate aminotransferase, mitochondrial | GOT2 | 2 | -3.199006939 | 0.000203 |
|  | K7ERI9 | Isoform of P02654, Apolipoprotein C-I (Fragment) | APOC1 | 2 | -3.240793717 | 1.87E-07 |
|  | O00151 | PDZ and LIM domain protein 1 | PDLIM1 | 3 | -3.267827988 | 4.29E-05 |
|  | P52907 | F-actin-capping protein subunit alpha-1 | CAPZA1 | 2 | -3.635709255 | 7.31E-08 |
| 3. LONGITUDINAL STUDY (Early vs Late time points): ALS-Slow | | | | | | |
| **REGULATION** | **Protein ID** | **Protein Descriptions** | **Gene Name** | **Peptide number** | **Log 2 FC_early _Slow/Late Slow** | **adjusted P.Value** |
| Up-regulated in the late time point ALS-Slow, top ten proteins identified by 2 or more peptides. | P10643 | Complement component C7 | C7 | 4 | 3.01663057 | 9.04E-10 |
|  | P15169 | Carboxypeptidase N catalytic chain | CPN1 | 3 | 2.859142315 | 8.93E-07 |
|  | P02656 | Apolipoprotein C-III | APOC3 | 3 | 2.78509104 | 8.62E-06 |
|  | P61158 | Actin-related protein 3 | ACTR3 | 2 | 2.616873098 | 3.62E-05 |
|  | P02765 | Alpha-2-HS-glycoprotein | AHSG | 8 | 2.53989109 | 8.57E-08 |
|  | P02743 | Serum amyloid P-component | APCS | 3 | 2.343521654 | 1.12E-05 |
|  | P62937 | Peptidyl-prolyl cis-trans isomerase A | PPIA | 4 | 2.273382133 | 0.000755 |
|  | P36955 | Pigment epithelium-derived factor | SERPINF1 | 3 | 2.14429825 | 8.90E-07 |
|  | G3V1A4 | Isoform of P23528, Cofilin 1 (Non-muscle), isoform CRA_a | CFL1 | 6 | 2.076603488 | 0.003942 |
|  | Q15582 | Transforming growth factor-beta-induced protein ig-h3 | TGFBI | 3 | 1.977209515 | 1.90E-05 |
| Down-regulated in the late time point for ALS-Slow, top ten proteins identified by 2 or more peptides. | P55072 | Transitional endoplasmic reticulum ATPase OS=Homo sapiens GN=VCP PE=1 SV=4 - [TERA_HUMAN] | VCP | 2 | -2.44303107 | 1.19E-06 |
|  | P63173 | 60S ribosomal protein L38 OS=Homo sapiens GN=RPL38 PE=1 SV=2 - [RL38_HUMAN] | RPL38 | 2 | -2.595924381 | 0.000255 |
|  | P62081 | 40S ribosomal protein S7 OS=Homo sapiens GN=RPS7 PE=1 SV=1 - [RS7_HUMAN] | RPS7 | 2 | -2.601730509 | 7.48E-09 |
|  | P07900 | Heat shock protein HSP 90-alpha OS=Homo sapiens GN=HSP90AA1 PE=1 SV=5 - [HS90A_HUMAN] | HSP90AA1 | 3 | -2.666824262 | 6.44E-07 |
|  | P52907 | F-actin-capping protein subunit alpha-1 OS=Homo sapiens GN=CAPZA1 PE=1 SV=3 - [CAZA1_HUMAN] | CAPZA1 | 2 | -2.829445108 | 5.27E-07 |
|  | P62805 | Histone H4 OS=Homo sapiens GN=HIST1H4A PE=1 SV=2 - [H4_HUMAN] | HIST1H4A | 5 | -2.924276504 | 2.36E-06 |
|  | K7ERI9 | Isoform of P02654, Apolipoprotein C-I (Fragment) OS=Homo sapiens GN=APOC1 PE=1 SV=1 - [K7ERI9_HUMAN] | APOC1 | 2 | -2.990208948 | 7.99E-05 |
|  | P48740-2 | Isoform of P48740, Isoform 2 of Mannan-binding lectin serine protease 1 OS=Homo sapiens GN=MASP1 - [MASP1_HUMAN] | MASP1 | 2 | -3.003721758 | 0.00123 |
|  | Q15365 | Poly(rC)-binding protein 1 OS=Homo sapiens GN=PCBP1 PE=1 SV=2 - [PCBP1_HUMAN] | PCBP1 | 2 | -3.024144625 | 0.000272 |
|  | O00479 | High mobility group nucleosome-binding domain-containing protein 4 OS=Homo sapiens GN=HMGN4 PE=1 SV=3 - [HMGN4_HUMAN] | HMGN4 | 2 | -3.487024396 | 0.007699 |
| MOUSE MODEL | | | | | | |
| 4. CROSS SECTIONAL STUDY (Fast vs Slow) pre-symptomatic time point, top 20 regulated proteins | | | | | | |
| **REGULATION** | **Protein ID** | **Protein Descriptions** | **Gene Name** | **Peptide number** | **Log 2 FC_Pre-Sympt _Fast/Slow** | **adjusted P.Value** |
| Up-regulated in the pre-symptomatic time point (fast genetic background), top ten proteins identified by 2 or more peptides. | P02535-3 | Isoform 3 of Keratin, type I cytoskeletal 10 | Krt10 | 3 | 5.079891057 | 0.002746 |
|  | P01863 | Ig gamma-2A chain C region, A allele | Ighg | 3 | 3.730982881 | 5.84E-06 |
|  | P02089 | Hemoglobin subunit beta-2 | Hbb-b2 | 22 | 3.697545209 | 1.22E-06 |
|  | P23953 | Carboxylesterase 1C | Ces1c | 7 | 3.412612381 | 0.00097 |
|  | P02088 | Hemoglobin subunit beta-1 | Hbb-b1 | 5 | 3.268452952 | 1.73E-07 |
|  | P12246 | Serum amyloid P-component | Apcs | 9 | 3.153123975 | 7.84E-07 |
|  | O55042-2 | Isoform 2 of Alpha-synuclein | Snca | 4 | 2.981802009 | 2.12E-05 |
|  | P07759 | Serine protease inhibitor A3K | Serpina3k | 6 | 2.366245045 | 0.005039 |
|  | Q9JHU9 | Inositol-3-phosphate synthase 1 | Isyna1 | 2 | 1.978232031 | 1.86E-05 |
|  | K3W4Q8 | Basigin | Bsg | 5 | 1.769481009 | 1.30E-05 |
| Down-regulated in the pre-symptomatic time point (fast genetic background), top ten proteins identified by 2 or more peptides. | A0A0A6YXN4 | Protein Ighv1-18 (Fragment) | Ighv1-18 | 3 | -2.193988066 | 5.62E-05 |
|  | A0A075B5T2 | Protein Ighv6-3 (Fragment) | Ighv6-3 | 3 | -2.263468959 | 0.000957 |
|  | A8DUK4 | Beta-globin | Hbbt1 | 2 | -2.338753792 | 0.000425 |
|  | P00329 | Alcohol dehydrogenase 1 | Adh1 | 3 | -2.367085863 | 4.58E-08 |
|  | Q91Y97 | Fructose-bisphosphate aldolase B | Aldob | 7 | -2.58417311 | 7.84E-07 |
|  | F8VQ07 | Protein 5830473C10Rik | 5830473C10Rik | 2 | -2.75789044 | 2.89E-05 |
|  | A0A075B5U7 | Protein Ighv1-22 (Fragment) | Ighv1-22 | 3 | -2.97546567 | 2.51E-05 |
|  | A0A075B6A3 | Protein Igha (Fragment) | Igha | 3 | -3.223071155 | 2.12E-05 |
|  | A0A075B5M7 | Protein Igkv5-39 | Igkv5-39 | 3 | -5.172094984 | 3.84E-08 |
|  | A0A0A6YY53 | Protein Ighg2c (Fragment) | Ighg2c | 2 | -5.983129049 | 4.58E-08 |
| 5. CROSS SECTIONAL STUDY ( Fast vs Slow) symptomatic time point, top 20 regulated proteins | | | | | | |
| **REGULATION** | **Protein ID** | **Protein Descriptions** | **Gene Name** | **Peptide number** | **Log 2 FC_Sympt _Fast/Slow** | **adjusted P.Value** |
| Up-regulated in the symptomatic time point (fast genetic background), top ten proteins identified by 2 or more peptides. | P02535-3 | Isoform 3 of Keratin, type I cytoskeletal 10 | Krt10 | 3 | 3.940646425 | 0.017741 |
|  | P02088 | Hemoglobin subunit beta-1 | Hbb-b1 | 5 | 3.810801203 | 1.90E-08 |
|  | P02089 | Hemoglobin subunit beta-2 | Hbb-b2 | 22 | 3.710006751 | 1.59E-06 |
|  | P12246 | Serum amyloid P-component | Apcs | 9 | 3.665287755 | 7.43E-08 |
|  | P01863 | Ig gamma-2A chain C region, A allele | Ighg | 3 | 3.663916884 | 8.47E-06 |
|  | P23953 | Carboxylesterase 1C | Ces1c | 7 | 2.987765988 | 0.003473 |
|  | P56400 | Platelet glycoprotein Ib beta chain | Gp1bb | 2 | 2.518031549 | 3.44E-06 |
|  | P39688;P39688-2 | Tyrosine-protein kinase Fyn | Fyn | 2 | 2.201724496 | 2.73E-05 |
|  | Q9D892 | Inosine triphosphate pyrophosphatase | Itpa | 2 | 2.01764677 | 0.00084 |
|  | A0A075B5R5 | Protein Ighv4-1 (Fragment) | Ighv4-1 | 2 | 1.956913766 | 0.002337 |
| Down-regulated in the symptomatic time point (fast genetic background), top ten proteins identified by 2 or more peptides. | A0A075B5M7 | Protein Igkv5-39 | Igkv5-39 | 3 | -2.708134857 | 0.00052 |
|  | A0A075B5R2 | Protein Ighv7-3 (Fragment) | Ighv7-3 | 3 | -2.773335817 | 0.010093 |
|  | P21550 | Beta-enolase | Eno3 | 5 | -2.872700842 | 0.000578 |
|  | P07310 | Creatine kinase M-type | Ckm | 4 | -3.012196237 | 0.007209 |
|  | A0A075B5U7 | Protein Ighv1-22 (Fragment) | Ighv1-22 | 3 | -3.365078639 | 4.43E-06 |
|  | A0A0R4J0I1 | MCG1051009 | Serpina3k | 7 | -3.493274742 | 0.007866 |
|  | A0A075B6A3 | Protein Igha (Fragment) | Igha | 3 | -3.656486744 | 3.86E-06 |
|  | P02798 | Metallothionein-2 | Mt2 | 2 | -3.804628909 | 0.000336 |
|  | P16015 | Carbonic anhydrase 3 | Ca3 | 2 | -4.0491561 | 0.002128 |
|  | A0A0A6YY53 | Protein Ighg2c (Fragment) | Ighg2c | 2 | -5.039773933 | 1.13E-06 |
| 6. LONGITUDINAL STUDY (Early Vs Late) fast progressing (129Sv) | | | | | | |
| **REGULATION** | **Protein ID** | **Protein Descriptions** | **Gene Name** | **Peptide number** | **Log 2 FC_Pre-Sympt _Fast/symptomatic Fast** | **adjusted P.Value** |
| Up-regulated in the symptomatic time point (129SV-FAST), top ten proteins identified by 2 or more peptides. | P12787 | Cytochrome c oxidase subunit 5A, mitochondrial | Cox5a | 2 | 4.031352604 | 0.002788 |
|  | A0A140T8M7 | Protein Rpl23a-ps3 | A0a140t8m7 | 5 | 2.81313878 | 1.11E-05 |
|  | Q6GQT9 | Nodal modulator 1 | Nomo1 | 2 | 2.55649849 | 6.97E-05 |
|  | Q9CQV8-2 | Isoform Short of 14-3-3 protein beta/alpha | 1433b | 3 | 2.497024996 | 0.000556 |
|  | P11438 | Lysosome-associated membrane glycoprotein 1 | Lamp1 | 3 | 2.394604609 | 1.38E-07 |
|  | F6YVP7 | Protein Gm10260 | F6yvp7 | 2 | 2.317644076 | 7.95E-05 |
|  | A0A140T8T3 | Protein Rps18-ps3 | A0a140t8t3 | 2 | 2.316944753 | 7.95E-05 |
|  | O54941 | SWI/SNF-related matrix-associated actin-dependent regulator of chromatin subfamily E member 1 | Smce1 | 2 | 2.278966655 | 0.011355 |
|  | Q6W8Q3 | Purkinje cell protein 4-like protein 1 | Pc4l1 | 2 | 2.153299568 | 0.001029 |
|  | O88569 | Heterogeneous nuclear ribonucleoproteins A2/B1 | Roa2 | 2 | 2.143454066 | 0.000601 |
| Down-regulated in the symptomatic time point (129SV, FAST), top ten proteins identified by 2 or more peptides. | Q8R5A3 | Amyloid beta A4 precursor protein-binding family B member 1-interacting protein | Ab1ip | 4 | -1.409254226 | 0.005492 |
|  | O88487 | Cytoplasmic dynein 1 intermediate chain 2 | Dc1i2 | 3 | -1.420554505 | 0.001684 |
|  | Q80UR4 | Mast cell protease-11 | Q80ur4 | 2 | -1.437063374 | 0.001841 |
|  | Q8VBT0 | Thioredoxin-related transmembrane protein 1 | Tmx1 | 2 | -1.439008301 | 0.000804 |
|  | D3YX85 | Arf-GAP with SH3 domain, ANK repeat and PH domain-containing protein 2 | D3yx85 | 2 | -1.628082986 | 0.034303 |
|  | Q7TN29 | Stromal membrane-associated protein 2 | Smap2 | 2 | -1.895845368 | 0.000126 |
|  | Q9CWL8 | Beta-catenin-like protein 1 | Ctbl1 | 2 | -2.026658185 | 7.95E-05 |
|  | Q8K4F0 | CD226 antigen | Cd226 | 3 | -2.028742107 | 0.002363 |
|  | Q9CXW3 | Calcyclin-binding protein | Cybp | 2 | -2.115578413 | 0.000931 |
|  | Q9D7S7-2 | Isoform 2 of 60S ribosomal protein L22-like 1 | Rl22l | 2 | -2.895491152 | 0.002879 |
| 7. Longitudinal Study ( Early Vs Late) Slow Progressing (C57) | | | | | | |
| **REGULATION** | **Protein ID** | **Protein Descriptions** | **Gene Name** | **Peptide number** | **Log 2 FC_Pre-Sympt _slow/symptomatic Slow** | **adjusted P.Value** |
| Up-regulated symptomatic time point (C57 SLOW), top ten proteins identified by 2 or more peptides. | F6YVP7 | Protein Gm10260 | F6yvp7 | 2 | 3.267528106 | 1.09E-06 |
|  | A0A140T8T3 | Protein Rps18-ps3 | A0a140t8t3 | 2 | 3.266633451 | 1.09E-06 |
|  | P41105 | 60S ribosomal protein L28 | Rl28 | 2 | 2.956534632 | 9.42E-05 |
|  | A0A140T8M7 | Protein Rpl23a-ps3 | A0a140t8m7 | 5 | 2.764896647 | 7.30E-06 |
|  | P12787 | Cytochrome c oxidase subunit 5A, mitochondrial | Cox5a | 2 | 2.462828572 | 0.042048 |
|  | Q8C5H8-2 | Isoform 2 of NAD kinase 2, mitochondrial | Nakd2 | 2 | 2.425133332 | 0.000275 |
|  | P35979 | 60S ribosomal protein L12 | Rl12 | 5 | 2.207004089 | 8.00E-05 |
|  | P11438 | Lysosome-associated membrane glycoprotein 1 | Lamp1 | 3 | 2.201897438 | 5.70E-07 |
|  | Q9D892 | Inosine triphosphate pyrophosphatase | Itpa | 2 | 2.185505148 | 0.002384 |
|  | P27659 | 60S ribosomal protein L3 | Rl3 | 4 | 2.16180411 | 9.43E-06 |
| Down-regulated in symptomatic time point (C57 SLOW), top ten proteins identified by 2 or more peptides. | Q8VDW0 | ATP-dependent RNA helicase DDX39A | Dx39a | 3 | -1.698416938 | 0.000232 |
|  | P21550 | Beta-enolase | Enob | 5 | -1.712730489 | 0.000207 |
|  | E9PV24 | Fibrinogen alpha chain | Fiba | 52 | -1.780547159 | 0.000528 |
|  | Q9DC51 | Guanine nucleotide-binding protein G(k) subunit alpha | Gnai3 | 3 | -1.824992077 | 0.000399 |
|  | P70663 | SPARC-like protein 1 | Sprl1 | 2 | -2.019280841 | 5.27E-05 |
|  | Q7TN29 | Stromal membrane-associated protein 2 | Smap2 | 2 | -2.05845991 | 3.10E-05 |
|  | Q9D7S7-2 | Isoform 2 of 60S ribosomal protein L22-like 1 | Rl22l | 2 | -2.328627996 | 0.009901 |
|  | P02798 | Metallothionein-2 OS=Mus musculus GN=Mt2 PE=1 SV=2 - [MT2_MOUSE] | Mt2 | 2 | -2.566294814 | 0.018964 |
|  | Q9CXW3 | Calcyclin-binding protein OS=Mus musculus GN=Cacybp PE=1 SV=1 - [CYBP_MOUSE] | Cybp | 2 | -2.873339468 | 2.47E-05 |
|  | D3YX85 | Arf-GAP with SH3 domain, ANK repeat and PH domain-containing protein 2 OS=Mus musculus GN=Asap2 PE=1 SV=2 - [D3YX85_MOUSE] | D3yx85 | 2 | -3.973545535 | 1.32E-05 |

**Supplementary Table 2. Functional analysis of the animal model proteomic data**. Only the top 10 enriched pathways and biological processes for each analysis are shown. 1) Pathway enrichment for pre-symptomatic stage and symptomatic stage in the cross-sectional study. 2) Enriched biological processes in the cross-sectionl study for pre-symptomatic and symptomatic stage 3) Pathway enrichment in the longitudinal study for the Slow 57 and for the Fast 129Sv genetic background. 4) Biological process enrichment in the longitudinal study for the for Slow 57 and for Fast 129 Sv genetic background.

| 1. CROSS-SECTIONAL STUDY (Fast 129 Sv versus Slow C57 progressing animal model)   PATHWAY ENRICHMENT | | |
| --- | --- | --- |
| Pre-symptomatic Stage | | |
| Term | P value | matched Genes |
| Glycolysis [Uniprot] | 6.18E-05 | Aldoa;Aldoart2;Pklr;Aldoc;Gapdh;Eno1;Eno2;Tpi1;Eno3;Pkm;Aldob;Aldoart1;Pfkp;Gm3839 |
| Intraflagellar transport [Reactome] | 0.000106884 | Tubb1;Dynll1;Tuba4a;Tuba1a;Tubb4b;Tuba1c;Tubb2a;Tubb6;Tubb2b;Tubb4a;Tubb3 |
| Gene Expression [Reactome] | 0.000143437 | Snw1;Dhx9;Gm17669;Usp7;Supt16;Gm5422;Eftud2;Psmd4;Hnrnph1;Csnk2a2;Sfn;Hnrnpa2b1;Psmc3;Hsp90aa1;Rpl7a;Rpl7;Eif3a;Psmd7;Snrpb;Prkcd;Prdx1;Rpl28;Hnrnpa1;Ppm1a;Pcbp1;Eif4a1;Ube2d1;Rpl26;Rpl27;Ywhag;Rps7;Psmc6;Rps4x;Rpl18a;Rps6;Hist1h4a; Hist1h4b; Hist1h4c; Hist1h4d; Hist1h4f; Hist1h4h; Hist1h4i; Hist1h4j; Hist1h4k; Hist1h4m; Hist2h4a; Hist4h4;Rps25;Tceb2;Rpl30;Ywhaz;Ywhah;Hnrnph2;Psme2;Sf3b2;Rpl10a;Pcbp2;Pabpc2;Snrpa;Ppp2r2a;Xpo1;Poldip3;Eif4b;Eif1ax;Hist3h2bb;Sf3a1;Psmd2;Dcp1a;Smarca5;Nxf1;Rps21;Rpl11;Eif3k;Ranbp2;Psmd13;Hnrnpf |
| Translocation of GLUT4 to the plasma membrane [Reactome] | 0.00063984 | Tubb1;Sfn;Rab8a;Rab4a;Rab10;Ywhag;Calm1; Calm2; Calm3;Rab11a;Ywhaz;Tuba4a;Tuba1a;Tubb4b;Tuba1c;Ywhah;Tubb2a;Myh9;Rab14;Tubb6;Tubb2b;Tubb4a;Tubb3;Tuba8 |
| Initial triggering of complement [Reactome] | 0.000798013 | Ighg2b;Ighg1;Ighg2c;Gm20547;C3;C4b;C1qb;C1qa;C1ra |
| Assembly of the primary cilium [Reactome] | 0.000814802 | Tubb1;Dync1i2;Hsp90aa1;Prkar2b;Rab8a;Actr1a;Ywhag;Rab11a;Pafah1b1;Dynll1;Tuba4a;Tuba1a;Tubb4b;Tuba1c;Tubb5;Cep162;Tubb2a;Tubb6;Tubb2b;Tubb4a;Tubb3 |
| Antigen Presentation: Folding, assembly and peptide loading of class I MHC [Reactome] | 0.000961042 | B2m;H2-Q10;H2-D1;H2-K1;H2-T23;Pdia3;Tap2;Sec31a;Sec24b;H2-Q4;Sar1b;Erap1 |
| Anchoring of the basal body to the plasma membrane [Reactome] | 0.001613369 | Dync1i2;Hsp90aa1;Prkar2b;Rab8a;Actr1a;Ywhag;Rab11a;Pafah1b1;Dynll1;Tuba4a;Tuba1a;Tubb4b;Tubb5;Cep162;Tubb4a |
| RHO GTPases activate PKNs [Reactome] | 0.002320882 | Sfn;Ywhag;Ppp1cb;Rhob;Hist1h4a; Hist1h4b; Hist1h4c; Hist1h4d; Hist1h4f; Hist1h4h; Hist1h4i; Hist1h4j; Hist1h4k; Hist1h4m; Hist2h4a; Hist4h4;Ywhaz;Ywhah;Pkn1;Rhoc;4930544G11Rik |
| Vesicle-mediated transport [Reactome] | 0.002979198 | Tubb1;Hbbt1;Gm20425;Hbb-bs;Golgb1;Sec23ip;Sec22b;Rab33b;Sfn;Dync1i2;Cops3;Cops4;Stam2;Ap2a2;Ap1g1;Rab6a;Eps15;Rab8a;Rab4a;Arpc4;Rab10;Actr2;Ywhag;Calm1; Calm2; Calm3;Rab11a;Pafah1b1;Ywhaz;Dynll1;Tuba4a;Tuba1a;Tubb4b;Tuba1c;Ywhah;Apoa1;Aak1;Sec31a;Clint1;Tsg101;Pafah1b2;Ocrl;Dync1li2;Tubb2a;Sec24b;Arrb1;Rab43;Mcfd2;Cops8;Myh9;Rab14;Hba-a2;Snx9;Hpx;Arrb2;Tf;Tubb6;Rab30;Arfgap2;Adrbk1;Arpc5;Vps25;Sar1b;Ykt6;Tubb2b;Snx2;Rab1b;Tubb4a;Chmp5;Chmp4b;Tubb3;Copb1;Tuba8;Hyou1;Copg1;Cog1;Tfg;Uso1 |
| Symptomatic stage | | |
| Term | P value | Matched Genes |
| Glycolysis [Reactome] | 9.39E-05 | Aldoart2;Pklr;Aldoc;Gpi;Gapdh;Eno1;Eno2;Pkm;Aldob;Aldoart1;Pgam1;Gm3839;Gm11214 |
| RHO GTPases activate PKNs [Reactome] | 0.00020299 | Sfn;Ywhag;Ppp1cb;Ywhae;Rhob;Ywhaz;Ywhah;Pkn1;Rhoc;4930544G11Rik |
| Gluconeogenesis [Reactome] | 0.000676159 | Aldoart2;Aldoc;Got1;Gpi;Gapdh;Eno1;Eno2;Aldob;Aldoart1;Slc25a11;Pgam1;Gm3839;Gm11214 |
| Activation of BH3-only proteins [Reactome] | 0.000977471 | Sfn;Ywhag;Ywhae;Ywhaz;Dynll1;Ywhah |
| Antigen processing: Ubiquitination & Proteasome degradation [Reactome] | 0.001182917 | Psmc1;Huwe1;Psmd6 |
| Activation of NF-kappaB in B cells [Reactome] | 0.001498386 | Psmc1;Psmd6 |
| Assembly of the primary cilium [Reactome] | 0.00219737 | Tubb1;Dync1i2;Rab8a;Ywhag;Ywhae;Rab11a;Dynll1;Tubb4b;Tubb5;Cep162;Ppp2r1a;Tubb2a;Tubb6;Tubb2b;Tubb4a;Tubb3;Dctn3 |
| Mitotic G1-G1/S phases [Reactome] | 0.002886155 | Pcna;Psmc1;Ppp2r1a;Psmd6 |
| Cholesterol biosynthesis [Reactome] | 0.003127144 | Gm43738;Acat3;Acat2;Hmgcs1;Fdps;Ggps1 |
| Intraflagellar transport [Reactome] | 0.003888578 | Tubb1;Dynll1;Tubb4b;Tubb2a;Tubb6;Tubb2b;Tubb4a;Tubb3 |
| 1. CROSS-SECTIONAL STUDY (Fast 129Sv versus Slow C57 progressing animal model)   BIOLOGICAL PROCESS ENRICHMENT: | | |
| Pre-symptomatic stage | | |
| Term | P value | Matched Genes |
| GO:0006958:complement activation, classical pathway | 0.001132153 | C3;C4b;Iglc2;Iglc3;Ighg;Ighm;C5;C1qb;Ighv3-6;Mbl2;Serping1;C1qa;C1sa;C1ra |
| GO:0007264:small GTPase mediated signal transduction | 0.001348134 | Gm20425;Rab33b;Rab3d;Rab6a;Rab12;Rab22a;Rab11b;Rab8a;Rab10;Rab8b;Arf3;Rab6b;Rab11a;Rhob;Rap1a;Rab3a;Arf5;Rac2;Arhgap1;Rhoc;Rab35;Rab39b;Rab43;Rab38;Rab14;Rab4b;Rab31;Rab30;Rap1b;Rab32;Rab1b;Rasgrp2 |
| GO:0007017:microtubule-based process | 0.002188326 | Tubb1;Vbp1;Pafah1b1;Tuba4a;Tuba1a;Tubb4b;Tuba1c;Tubb5;Tubb2a;Tubb6;Tubb2b;Tubb4a;Tubb3 |
| GO:0006457:protein folding | 0.003051315 | Gm5160;Nudc;P4hb;Hsp90ab1;Ppia;Pdia3;Hspa4l;Vbp1;Cct4;Tubb5;Pfdn6;Aars;Qsox1;Tmx3;Tbcd;Ero1a;Tmx1;Pdia6;Dnajb11;Nudcd2;Trap1;Ppid;Erp44;Dnajb4;Mesdc2;Ranbp2 |
| GO:0006509:membrane protein ectodomain proteolysis | 0.004289702 | Adam10;Prkcq;Myh9;Rbmxl1;Erap1;Rbmx |
| GO:0051897:positive regulation of protein kinase B signaling | 0.004722143 | Angpt1;Ilk;F10;Thbs1;Hcls1;Hpse;Mtdh;Arrb2;Stk3 |
| GO:0034097:response to cytokine | 0.005760881 | Itih4;Ddost;Serpina1a;Serpina3k;Serpina1b;Serpina1d;Serpina1e;Serpina3m;Acp5;Aldh1a2;Serpina3n |
| GO:0043434:response to peptide hormone | 0.011132271 | Atp2a2;Serpina1a;Serpina3k;Nefl;Col1a1;Serpina1b;Serpina1d;Serpina1e;Serpina3m;Serpina3n |
| GO:0000226:microtubule cytoskeleton organization | 0.012056791 | Nefl;Gapdh;Pafah1b1;Dync1li2;Tbcd;Map1s;Tuba8 |
| GO:0006910:phagocytosis, recognition | 0.012138367 | Iglc2;Iglc3;Ighg;Ighm;Ighv3-6 |
| Symptomatic stage | | |
| Term | P value | Matched Genes |
| GO:0034097:response to cytokine | 4.65E-06 | Itih4;Coro1a;Serpina3k;Col3a1;Serpina1b;Serpina3c;Mapkapk2;Serpina1d;Serpina1e;Serpina3m;Pml;Aldh1a2;Serpina3n |
| GO:0006508:proteolysis | 0.001039677 | F10;Ctsg;Ctsh;Cps1;Erap1 |
| GO:0042752:regulation of circadian rhythm | 0.001175917 | Ube3a;Ppp1ca;Ppp1cb;Ppp1cc;Rock2;Creb1;Pml;Pspc1;Sfpq |
| GO:0006953:acute-phase response | 0.002341374 | Itih4;Orm2;Crp;Serpina1b;Stat3;Orm1;Hp;Serpina3n;Plscr1 |
| GO:0006910:phagocytosis, recognition | 0.003215876 | Iglc2;Iglc3;Ighg;Ighm;Ighv3-6 |
| GO:0070936:protein K48-linked ubiquitination | 0.003215876 | Ube2d4;Ube3a;Ube2g2;Ube2d1;Ube2k |
| GO:0006096:glycolytic process | 0.004900046 | Aldoart2;Aldoc;Gpi;Gapdh;Eno1;Eno2;Pkm;Aldob;Pgam1;Gm3839 |
| GO:0007010:cytoskeleton organization | 0.006127602 | Sptb;Tpm2;Rac1;Tubb4b;Ywhah;Sptbn1;Tubb2a;Dock2;Tubb6;Tubb2b;Tubb4a;Tubb3;Rhoa |
| GO:0006509:membrane protein ectodomain proteolysis | 0.009108796 | Adam10;Prkcq;Rbmxl1;Erap1;Rbmx |
| GO:0036120:cellular response to platelet-derived growth factor stimulus | 0.009108796 | Rdx;Fyn;Creb1;Iqgap1;Coro1b |
| 3. LONGITUDINAL STUDY (Early versus Late time point)  PATHWAY ENRICHMENT | | |
| SLOW C57 | | |
| Term | P value | Matched Genes |
| RHO GTPases activate PKNs [Reactome] | 2.22E-05 | Sfn;Ywhag;Ppp1cb;Ywhae;Rhob;Hist1h4a; Hist1h4b; Hist1h4c; Hist1h4d; Hist1h4f; Hist1h4h; Hist1h4i; Hist1h4j; Hist1h4k; Hist1h4m; Hist2h4a; Hist4h4;Ywhaz;Ywhah;Pkn1;Hist1h3b; Hist1h3c; Hist1h3d; Hist1h3e; Hist1h3f; Hist2h3b; Hist2h3c1; Hist2h3c2;Rhoc;Ppp1r14a;4930544G11Rik |
| Programmed Cell Death [Reactome] | 4.46E-05 | Dffa;Nmt1;Sfn;Lmnb1;Sptan1;Vim;Lmna;Ywhag;Ywhae;Cycs;Ywhaz;Hmgb1;Dynll1;Ywhah;Kpnb1;Rock1;Prkcq;Satb1;Kpna1;Bcap31;Dnm1l;Stk24;Dynll2;Diablo;Add1;Ripk3 |
| DNA Replication [Reactome] | 0.00020031 | Psmb6;Psmf1;Psmd5;Psmd2;Psmd6;Psmd13 |
| Glycolysis [Reactome] | 0.000347361 | Aldoart2;Pklr;Pgam2;Aldoc;Gpi;Pfkl;Gapdh;Eno1;Eno3;Pfkm;Pkm;Aldob;Aldoart1;Pgam1;Gm3839;Gm11214 |
| Gluconeogenesis [Reactome] | 0.000368921 | Aldoart2;Pcx;Pgam2;Aldoc;Gpi;Mdh2;Mdh1;Gapdh;Eno1;Eno3;Slc25a1;Aldob;Aldoart1;Slc25a11;Pgam1;Gm3839;Gm11214 |
| Mitotic G1-G1/S phases [Reactome] | 0.000424577 | Hdac1;Ppp2cb;Ppp2ca;Psmb6;Psmf1;Psmd5;Psmd2;Psmd6;Psmd13 |
| CLEC7A (Dectin-1) signaling [Reactome] | 0.000674461 | Nfkb1;Calm1; Calm2; Calm3;Psmb6;Psmf1;Psmd5;Psmd2;Psmd6;Psmd13 |
| glycolysis [Uniprot] | 0.000727412 | Aldoa;Aldoart2;Pklr;Aldoc;Gpi;Pfkl;Gapdh;Eno1;Eno3;Pfkm;Pkm;Aldob;Aldoart1;Gm3839 |
| Organelle biogenesis and maintenance [Reactome] | 0.000771648 | Dync1i2;Hsp90aa1;Sept2;Mapk14;Rab8a;Atp5b;Mrps6;Actr1a;Arf4;Ywhag;Ywhae;Rab11a;Cycs;Dynll1;Tuba4a;Tuba1a;Tubb4b;Tuba1c;Tubb5;Gabpa;Exoc5;Hcfc1;Cep162;Tubb2a;Tubb6;Tubb2b;Dynll2;Tubb4a;Mrpl12;Tubb3 |
| Translocation of GLUT4 to the plasma membrane [Reactome] | 0.000821295 | Sfn;Rab8a;Rab10;Ywhag;Calm1; Calm2; Calm3;Ywhae;Rab11a;Vamp2;Ywhaz;Tuba4a;Tuba1a;Tubb4b;Tuba1c;Ywhah;Exoc5;Tubal3;Stxbp3;Tubb2a;Lnpep;Myh9;Tubb6;Tubb2b;Tubb4a;Rab13;Tubb3;Tuba8 |
| FAST 129Sv | | |
| Term | P value | Matched Genes |
| Intraflagellar transport [Reactome] | 0.00013777 | Tubb1;Dynll1;Tuba4a;Tuba1a;Tubb4b;Tuba1c;Tubb2a;Tubb6;Tubb2b;Dynll2;Tubb4a;Tubb3 |
| S Phase [Reactome] | 0.000251486 | Psmb1;Mcm4;Psmc4;Psmc1;Psmd1;Rfc3;Psmd6 |
| Degradation of beta-catenin by the destruction complex [Reactome] | 0.000584131 | Psmb1;Psmc4;Psmc1;Ppp2ca;Psmd1;Psmd6 |
| Translocation of GLUT4 to the plasma membrane [Reactome] | 0.000741196 | Tubb1;Sfn;Akt1;Rab10;Ywhag;Ywhae;Rac1;Ywhaz;Tuba4a;Tuba1a;Tubb4b;Tuba1c;Ywhah;Tubal3;Akt2;Tubb2a;Prkaa2;Lnpep;Tubb6;Tubb2b;Exoc2;Tubb4a;Rab13;Tubb3;Tuba8 |
| Negative epigenetic regulation of rRNA expression [Reactome] | 0.001092708 | Hdac1;Sap18;H2afz;Hist1h2bm;H2afx;Hist1h2ba;Hist1h3b; Hist1h3c; Hist1h3d; Hist1h3e; Hist1h3f; Hist2h3b; Hist2h3c1; Hist2h3c2;Rbbp4;Rbbp7;Hist2h2ab;Hist3h2bb |
| CD209 (DC-SIGN) signaling [Reactome] | 0.00139425 | Nras;Nfkb1;Lyn;Icam2;Fyn;Prkacb;Pak2;Raf1 |
| Thrombin signalling through proteinase activated receptors (PARs) [Reactome] | 0.00139425 | F2;Gnaq;Gnb1;Mapk1;Gng2;Mapk3;Arrb1;Arrb2 |
| Mitotic G1-G1/S phases [Reactome] | 0.001644385 | Psmb1;Hdac1;Mcm4;Psmc4;Psmc1;Ppp2ca;Psmd1;Rbbp4;Psmd6 |
| DNA Replication [Reactome] | 0.001906935 | Psmb1;Mcm4;Psmc4;Psmc1;Psmd1;Rfc3;Psmd6 |
| Intrinsic Pathway for Apoptosis [Reactome] | 0.002499205 | Sfn;Casp8;Ywhag;Ywhae;Ywhaz;Dynll1;Ywhah;Casp3;Dynll2;Diablo |
| 4. BIOLOGICAL PROCESS ENRICHMENT | | |
| SLOW C57 | | |
| Term | P value | Matched Genes |
| GO:0007010:cytoskeleton organization | 0.000625328 | Thy1;Krt4;Sptb;Cfl1;Lsp1;Tpm2;Tuba4a;Tuba1a;Tubb4b;Tuba1c;Ywhah;Tubal3;Sptbn1;Tubb2a;Tubb6;Pstpip2;Tubb2b;Tubb4a;Tubb3;Rhoa |
| GO:0006412:translation | 0.002977923 | Rpl23a-ps3;Rps18-ps3;Gm10260;Rpl21;Rpl7a;Rps16;Rpl7;Rps2;Rpl3;Rpl12;Rpl28;Rpl5;Rpl13;Slc25a4;Slc25a5;Rps20;Rpl27;Rps15a;Rps23;Rps11;Rps13;Rps4x;Rpl23;Rps26;Rps3;Rpl32;Rps27a;Eif5b;Slc25a31;Rps27l;Slc25a1;Aimp2;Rpl14;Slc25a11;Rpl11;Rpl4;Mrpl12 |
| GO:0006096:glycolytic process | 0.00365953 | Aldoart2;Pgam2;Aldoc;Gpi;Pfkl;Gapdh;Eno1;Eno3;Pfkm;Pkm;Hkdc1;Aldob;Pgam1;Gm3839 |
| GO:0051258:protein polymerization | 0.004520147 | Fga;Nefl;Vtn;Sept2;Fgb;Fgg;Chmp2a |
| GO:0051290:protein heterotetramerization | 0.004520147 | Anxa2;S100a10;Hist1h4a; Hist1h4b; Hist1h4c; Hist1h4d; Hist1h4f; Hist1h4h; Hist1h4i; Hist1h4j; Hist1h4k; Hist1h4m; Hist2h4a; Hist4h4;Hist1h3b; Hist1h3c; Hist1h3d; Hist1h3e; Hist1h3f; Hist2h3b; Hist2h3c1; Hist2h3c2;Nup54;Farsa;Farsb |
| GO:0042384:cilium assembly | 0.006214731 | Sept2;Rab8a;Actr2;Exoc5;Ocrl;Cep162;Ehd3;Ehd1 |
| GO:0007017:microtubule-based process | 0.009864473 | Tuba4a;Tuba1a;Tubb4b;Tuba1c;Tubb5;Tubal3;Kif5b;Tubb2a;Tubb6;Tubb2b;Dynll2;Tubb4a;Tubb3 |
| GO:0000281:mitotic cytokinesis | 0.011025735 | Sept7;Cfl1;Stmn1;Myh10;Sptbn1;Chmp4b |
| GO:0010506:regulation of autophagy | 0.011025735 | Pip4k2a;Hmgb1;Pip4k2b;Cisd2;Chmp4b;Pycard |
| GO:0042026:protein refolding | 0.011025735 | B2m;Hsp90aa1;Hspa1l;Hspa2;Hspa8;Hspd1 |
| FAST 129Sv | | |
| Term | P value | Matched Genes |
| GO:0007017:microtubule-based process | 4.38E-06 | Tubb1;Pafah1b1;Tuba4a;Tuba1a;Tubb4b;Tuba1c;Tubb5;Tubal3;Kif5b;Tubb2a;Tubb6;Dctn2;Tubb2b;Dynll2;Tubb4a;Tubb3 |
| GO:0010501:RNA secondary structure unwinding | 7.81E-05 | Eif4a2;Ddx6;Eif4a1;Ddx17;Ddx46;Ddx5;Ddx3x;Ago2;Ddx39a;Ddx1;Ddx21;Ddx39b |
| GO:0034976:response to endoplasmic reticulum stress | 0.000313197 | Atp2a2;Pdia4;P4hb;Hspa5;Pdia3;Thbs1;Ptpn1;Ufm1;Fam129a;Ufl1;Eif2b5;Ero1a;Tmx1;Tardbp;Pdia6;Ufc1;Erp44;Hyou1 |
| GO:0006412:translation | 0.000392921 | Rpl36-ps3;Rpl23a-ps3;Rps18-ps3;Gm10260;Rpl21;Rpl7a;Rps16;Rpsa;Rpl3;Rpl12;Rpl28;Rpl5;Rpl13;Slc25a4;Slc25a5;Rps20;Rpl27;Rpl37a;Rps7;Rps15a;Rps23;Rps11;Rps13;Rps26;Slc25a31;Rps27l;Slc25a12;Slc25a1;Aimp2;Rps21;Slc25a11;Nhp2;Rps19;Slc25a20 |
| GO:0046777:protein autophosphorylation | 0.000853196 | Cad;Stk10;Lyn;Pdgfra;Flt4;Fyn;Syk;Mapkapk2;Map4k1;Alk;Aak1;Map3k3;Jak2;Trim28;Stk17b;Prkd2;Stk24;Ripk3;Htatip2 |
| GO:0007010:cytoskeleton organization | 0.001512245 | Krt4;Sptb;Cfl1;Tpm2;Tuba4a;Tuba1a;Tubb4b;Tuba1c;Ywhah;Tubal3;Sptbn1;Tubb2a;Tubb6;Tubb2b;Tubb4a;Tubb3;Rhoa |
| GO:0051017:actin filament bundle assembly | 0.001522909 | Ezr;Actn4;Cdc42;Pls1;Lcp1;Actn1;Pls3;Add2;Add1 |
| GO:0007155:cell adhesion | 0.002115491 | Prkca;Ache;Thbs1;Cd9;Itgav;Gp1bb;Rhob;Tgfbi;Thbs2;Col6a1;Lgals3bp;Cd84;Lamb3;Myh10;Azgp1;Prkd2;Myh9;Emilin1;Lpxn;Parvb;Rhoa |
| GO:0036120:cellular response to platelet-derived growth factor stimulus | 0.003537025 | Itgb3;Cbl;Rdx;Fyn;Creb1;Iqgap1 |
| GO:0071353:cellular response to interleukin-4 | 0.003651554 | Coro1a;Hsp90ab1;Rplp0;Hspa5;Impdh2;Rpl3;Tcf7;Pml;Arg1 |

**Supplementary Table 3.** Top regulated proteins in plasma from Wild type versus SOD1G93A transgenic mice at the pre-symptomatic and symptomatic stage of disease, for both genetic backgrounds under investigation (slow C57 and Fast 129Sv.

|  | Animal model: Wild type vs SOD1 | | | |
| --- | --- | --- | --- | --- |
|  | PRE-SYMPTOMATIC TIME POINT | | | |
| SLOW C57 |  | Protein_Descriptions | logFC_ALS/Control | p value |
|  | Top 10 upregulated proteins when comparing Pre-WTC57 Vs Pre-SOD1C5 | Protein Gm10260 | 3.651 | 2.59E-07 |
|  |  | Protein Rps18-ps3 | 3.650 | 2.59E-07 |
|  |  | 60S ribosomal protein L7a | 3.318 | 1.02E-06 |
|  |  | Isoform 2 of NAD kinase 2, mitochondrial | 2.901 | 3.53E-05 |
|  |  | 60S ribosomal protein L28 | 2.823 | 0.000118 |
|  |  | Protein Rpl23a-ps3 | 2.80 | 4.64E-06 |
|  |  | Transmembrane 7 superfamily member 3 | 2.75 | 9.49E-05 |
|  |  | Lysosome-associated membrane glycoprotein 1 | 2.41 | 1.24E-07 |
|  |  | Nuclear factor NF-kappa-B p105 subunit | 2.21 | 0.000335 |
|  |  | 40S ribosomal protein S20 | 2.12 | 9.75E-05 |
|  | TOP 10 down-regulated proteins when comparing Pre-WTC57 Vs Pre-SOD1C5 | N-acetylglucosamine-6-phosphate deacetylase | -2.94 | 0.002186 |
|  |  | Pleckstrin homology domain-containing family A member 2 | -2.35 | 1.78E-05 |
|  |  | Usher syndrome type-1C protein-binding protein 1 | -2.33 | 0.000746 |
|  |  | Arf-GAP with SH3 domain, ANK repeat and PH domain-containing protein 2 | -2.23 | 0.002244 |
|  |  | Calcyclin-binding protein | -2.15 | 0.000377 |
|  |  | Major facilitator superfamily domain-containing protein 2B | -1.93 | 0.001054 |
|  |  | Cytoplasmic protein NCK2 | -1.88 | 0.000249 |
|  |  | Splicing factor U2AF 35 kDa subunit | -1.87 | 0.009106 |
|  |  | U6 snRNA-associated Sm-like protein LSm7 | -1.81 | 0.007524 |
|  |  | Thrombospondin-1 | -1.70 | 2.94E-06 |
| FAST 129 SV |  | Protein_Descriptions | logFC_ALS/Control | p value |
|  | Top 10 upregulated proteins when comparing Pre-WTS  vs Pre-SOD1129Sv | 60S ribosomal protein L7a | 3.34 | 1.75E-06 |
|  |  | Protein Rpl23a-ps3 | 2.96 | 4.34E-06 |
|  |  | NADH dehydrogenase [ubiquinone] flavoprotein 3, mitochondrial | 2.52 | 5.92E-05 |
|  |  | Endonuclease domain-containing 1 protein | 2.48 | 0.005938 |
|  |  | Isoform Short of 14-3-3 protein beta/alpha | 2.39 | 0.000496 |
|  |  | Synaptojanin-1 | 2.292 | 0.004855 |
|  |  | 60S ribosomal protein L28 | 2.248 | 0.001192 |
|  |  | Lysosome-associated membrane glycoprotein 1 | 2.127 | 1.01E-06 |
|  |  | 60S ribosomal protein L22 | 2.125 | 0.001958 |
|  |  | Isoform 3 of Periostin | 2.11 | 0.001628 |
|  | Top 10 downregulated proteins when comparing Pre-WTSv  vs Pre-SOD1129Sv | DnaJ homolog subfamily C member 7 | -3.07 | 9.60E-05 |
|  |  | Isoform Cytoplasmic of Glutathione reductase, mitochondrial | -2.50 | 1.22E-05 |
|  |  | Isoform 4 of Putative RNA-binding protein Luc7-like 2 | -2.41 | 0.004474 |
|  |  | Endothelial cell-selective adhesion molecule | -2.35 | 2.74E-05 |
|  |  | Isoform 2 of ER membrane protein complex subunit 1 | -2.14 | 0.004855 |
|  |  | AP-1 complex subunit gamma-1 | -2.10 | 8.57E-05 |
|  |  | Cytoplasmic protein NCK2 | -2.06 | 0.000125 |
|  |  | Dynein light chain 2, cytoplasmic | -1.91 | 0.00051 |
|  |  | Casein kinase II subunit alpha OS=Mus musculus | -1.89 | 0.006308 |
|  |  | SH3 domain-binding glutamic acid-rich-like protein 3 * | -1.88 | 0.000398 |
|  |  |  |  |  |
|  | SYMPTOMATIC TIME POINT | | | |
| SLOW C57 |  | Protein_Descriptions | logFC_ALS/Control | p value |
|  | TOP 10 UP regulated proteins when comparing Sym-WTC57 Vs Sym-SOD1C5 | Barrier-to-autointegration factor | 2.855 | 0.001644 |
|  |  | CD2-associated protein | 2.445 | 0.000265 |
|  |  | Arf-GAP with SH3 domain, ANK repeat and PH domain-containing protein 2 | 2.416 | 0.001827 |
|  |  | Reticulocalbin-2 | 2.406 | 0.000613 |
|  |  | T-cell surface glycoprotein CD5 | 2.404 | 0.003497 |
|  |  | Non-lysosomal glucosylceramidase | 2.372 | 0.000576 |
|  |  | Calcyclin-binding protein | 2.34 | 0.000305 |
|  |  | Beta-catenin-like protein 1 | 2.079 | 6.82E-05 |
|  |  | Translation machinery-associated protein 7 | 2.07 | 0.002152 |
|  |  | Diacylglycerol kinase | 2.062 | 0.000452 |
|  | TOP 10 down regulated proteins when comparing Sym-WTC57 Vs Sym-SOD1C5 | Isoform 2 of Protein FAM63A | -2.41 | 0.00028 |
|  |  | ER membrane protein complex subunit 8 | -2.1496 | 0.00039 |
|  |  | Isoform 3 of Pre-mRNA 3-end-processing factor FIP1 | -2.07983 | 0.004131 |
|  |  | Purkinje cell protein 4-like protein 1 | -2.07469 | 0.001045 |
|  |  | Inosine triphosphate pyrophosphatase | -1.94067 | 0.005785 |
|  |  | Beta-globin | -1.73 | 0.007216 |
|  |  | 26S proteasome non-ATPase regulatory subunit 4 | -1.68 | 0.001168 |
|  |  | A-kinase anchor protein 5 | -1.595 | 0.001581 |
|  |  | Ancient ubiquitous protein 1 | -1.563 | 0.001464 |
|  |  | Thrombospondin-1 | -1.705 | 2.94E-06 |
| FAST 129SV |  | Protein_Descriptions | logFC_ALS/Control | p value |
|  | TOP 10 UP regulated proteins when comparing Sym-WTSv  Vs Sym-SOD1129Sv | Dual-specificity mitogen-activated protein kinase kinase 2 | 2.207 | 0.001094 |
|  |  | Diacylglycerol kinase | 2.026 | 0.000562 |
|  |  | Vesicle-associated membrane protein, associated protein B and C | 1.82 | 0.000442 |
|  |  | Alpha-1-antitrypsin 1-4 | 1.809 | 0.009774 |
|  |  | Beta-catenin-like protein 1 | 1.786 | 0.000313 |
|  |  | Platelet glycoprotein Ib beta chain | 1.770 | 1.66E-05 |
|  |  | Eukaryotic translation initiation factor 5 | 1.759 | 0.000378 |
|  |  | Calcyclin-binding protein | 1.749 | 0.004124 |
|  |  | Protein PRRC2C | 1.654 | 0.000562 |
|  |  | ATP-dependent RNA helicase DDX39A | 1.605 | 0.000562 |
|  | TOP 10 down regulated proteins when comparing Sym-WTSv  Vs Sym-SOD1129Sv | Carbonic anhydrase 3 | -3.2932 | 0.001302 |
|  |  | Creatine kinase M-type | -2.766 | 0.000854 |
|  |  | Protein S100-A6 | -2.307 | 0.000291 |
|  |  | ER membrane protein complex subunit 8 | -2.18 | 0.000386 |
|  |  | Nodal modulator 1 | -2.15 | 0.000358 |
|  |  | Isoform 3 of Pre-mRNA 3-end-processing factor FIP1 | -1.97 | 0.00813 |
|  |  | Creatine kinase S-type, mitochondrial | -1.97 | 0.000442 |
|  |  | Phosphoinositide phospholipase C | -1.88 | 0.005568 |
|  |  | Phospholipid scramblase 1 | -1.837 | 0.002692 |
|  |  | Isoform 2 of Calcium/calmodulin-dependent protein kinase type II subunit gamma | -1.802 | 0.003149 |

**Supplementary Table 4. Functional analysis for the proteomic study comparing wild type versus transgenic ALS SOD1G93A mice.** Pathway enrichment analysis were performed at pre-symptomatic and symptomatic stages of disease, for both genetic backgrounds, slow C57 and Fast 129Sv. Only the top 10 enriched pathways for each analysis are shown here.

|  | ALS VERSUS CONTROL | | |
| --- | --- | --- | --- |
|  | PATHWAY ENRICHMENT | | |
|  | PRE-SYMPTOMATIC TIME POINT | | |
| SLOW C57 | **term** | **pvalue** | **Matched Genes** |
|  | Assembly of the primary cilium | 1,64E-05 | Tubb1;Trip11;Hsp90aa1;Rab8a;Actr1a;Arf4;Ywhag;Ywhae;Rab11a;Pafah1b1;Dynll1;Tuba4a;Tuba1a;Tubb4b;Tuba1c;Tubb5;Mapre1;Cep162;Ppp2r1a;Tubb2a;Tubb6;Tubb2b;Dynll2;Exoc2;Tubb4a;Tubb3;Dync1h1;Ssna1;Arl3 |
|  | Organelle biogenesis and maintenance | 3,88E-05 | Tubb1;Trip11;Hsp90aa1;Mapk14;Rab8a;Atp5b;Actr1a;Arf4;Ywhag;Ywhae;Rab11a;Pafah1b1;Dynll1;Tuba4a;Tuba1a;Tubb4b;Tuba1c;Tubb5;Mapre1;Cep162;Ppp2r1a;Tubb2a;Prkaa2;Tubb6;Mrps36;Tubb2b;Dynll2;Exoc2;Tubb4a;Mrpl12;Tubb3;Dync1h1;Ssna1;Arl3 |
|  | Intraflagellar transport | 5,33E-05 | Tubb1;Trip11;Dynll1;Tuba4a;Tuba1a;Tubb4b;Tuba1c;Tubb2a;Tubb6;Tubb2b;Dynll2;Tubb4a;Tubb3 |
|  | Anchoring of the basal body to the plasma membrane | 0,000549969 | Hsp90aa1;Rab8a;Actr1a;Ywhag;Ywhae;Rab11a;Pafah1b1;Dynll1;Tuba4a;Tuba1a;Tubb4b;Tubb5;Mapre1;Cep162;Ppp2r1a;Tubb4a;Dync1h1;Ssna1 |
|  | Post-translational protein modification | 0,001433571 | Hgs;Usp24;Golgb1;Hist1h2al;Sec24c;Psmb1;Psmd4;Psmc3;Spta1;Sptan1;Pcna;Muc13;Tsta3;Glb1;Pdia3;Mgat1;Plaur;Canx;Brcc3;Rad23a;Rad23b;Ruvbl1;Arf4;Sumo1;Dynll1;Ctsc;Psmd1;Sptb;Babam1;Sec31a;Usp9x;Trappc1;Pml;Sptbn1;Top2b;Hist2h2ab;Nop58;Galnt2;Snx3;Thbs1;Amdhd2;Psmd2;Psmd6;Nup155;4930544G11Rik;Smc3;Nup37;Lman1;Dynll2;Rab1b;Dync1h1;Trappc2l;Copg1;Psma4;Psma1;Uchl5;Preb;Uba2 |
|  | AURKA Activation by TPX2 | 0,001974605 | Hsp90aa1;Actr1a;Ywhag;Ywhae;Pafah1b1;Dynll1;Tuba4a;Tuba1a;Tubb4b;Tubb5;Mapre1;Ppp2r1a;Tubb4a;Dync1h1;Ssna1 |
|  | Loss of Nlp from mitotic centrosomes | 0,001974605 | Hsp90aa1;Actr1a;Ywhag;Ywhae;Pafah1b1;Dynll1;Tuba4a;Tuba1a;Tubb4b;Tubb5;Mapre1;Ppp2r1a;Tubb4a;Dync1h1;Ssna1 |
|  | Loss of proteins required for interphase microtubule organizationÂ from the centrosome | 0,001974605 | Hsp90aa1;Actr1a;Ywhag;Ywhae;Pafah1b1;Dynll1;Tuba4a;Tuba1a;Tubb4b;Tubb5;Mapre1;Ppp2r1a;Tubb4a;Dync1h1;Ssna1 |
|  | Activation of anterior HOX genes in hindbrain development during early embryogenesis | 0,002254128 | Kdm6a;Hdac3;H2afz;Hist1h2bm;H2afx;Hist1h4a; Hist1h4b; Hist1h4c; Hist1h4d; Hist1h4f; Hist1h4h; Hist1h4i; Hist1h4j; Hist1h4k; Hist1h4m; Hist2h4a; Hist4h4;Hist1h2ba;Hist1h3b; Hist1h3c; Hist1h3d; Hist1h3e; Hist1h3f; Hist2h3b; Hist2h3c1; Hist2h3c2;Rbbp4;Rbbp7;Hist2h2ab;Hist3h2bb |
|  | Activation of HOX genes during differentiation | 0,002254128 | Kdm6a;Hdac3;H2afz;Hist1h2bm;H2afx;Hist1h4a; Hist1h4b; Hist1h4c; Hist1h4d; Hist1h4f; Hist1h4h; Hist1h4i; Hist1h4j; Hist1h4k; Hist1h4m; Hist2h4a; Hist4h4;Hist1h2ba;Hist1h3b; Hist1h3c; Hist1h3d; Hist1h3e; Hist1h3f; Hist2h3b; Hist2h3c1; Hist2h3c2;Rbbp4;Rbbp7;Hist2h2ab;Hist3h2bb |
| FAST 129SV | **term** | **pvalue** | **Matched Genes** |
|  | ABC-family proteins mediated transport [Reactome] | 3,86E-06 | Psmb1;Psmd4;Psmd1;Psmb6;Abca7;Psma6 |
|  | Post-translational protein modification [Reactome] | 5,01E-06 | Tmed7;Usp7;Hist1h2al;Galnt4;Psmb1;Psmd4;Dpm1;Cope;Foxk1;Rad23a;Rad23b;Ube2d1;Arf4;Dynll1;Psmd1;Sptb;Usp9x;Trappc1;Psmb6;Pml;Hcfc1;Sptbn1;Top2b;Hist2h2ab;Dync1li2;Otub1;Galnt7;Yod1;Rpa1;Arrb2;Ubxn1;Nans;Dctn2;Ppp6c;4930544G11Rik;Smc3;Dynll2;Tmed10;Rab1b;Alg2;Ranbp2;Nup50;Uchl3;Trappc2l;Psma6;Copg1;Uchl5;Preb;Ctsz;Cog1;Tfg;Uba2;Uso1 |
|  | Organelle biogenesis and maintenance [Reactome] | 5,89E-06 | Tubb1;Trip11;Hsp90aa1;Prkar2b;Mapk14;Rab8a;Atp5b;Actr1a;Arf4;Ywhag;Ywhae;Rab11a;Pafah1b1;Dynll1;Tuba4a;Tuba1a;Tubb4b;Tuba1c;Tubb5;Exoc5;Mapre1;Hcfc1;Cep162;Ppp2r1a;Tubb2a;Prkaa2;Tubb6;Dctn2;Mrps36;Tubb2b;Dynll2;Exoc2;Tubb4a;Mrpl12;Tubb3;Ssna1 |
|  | Assembly of the primary cilium [Reactome] | 8,75E-06 | Tubb1;Trip11;Hsp90aa1;Prkar2b;Rab8a;Actr1a;Arf4;Ywhag;Ywhae;Rab11a;Pafah1b1;Dynll1;Tuba4a;Tuba1a;Tubb4b;Tuba1c;Tubb5;Exoc5;Mapre1;Cep162;Ppp2r1a;Tubb2a;Tubb6;Dctn2;Tubb2b;Dynll2;Exoc2;Tubb4a;Tubb3;Ssna1 |
|  | Orc1 removal from chromatin [Reactome] | 4,26E-05 | Psmb1;Psmd4;Psmd1;Psmb6;Psma6 |
|  | Regulation of DNA replication [Reactome] | 4,26E-05 | Psmb1;Psmd4;Psmd1;Psmb6;Psma6 |
|  | Removal of licensing factors from origins [Reactome] | 4,26E-05 | Psmb1;Psmd4;Psmd1;Psmb6;Psma6 |
|  | Switching of origins to a post-replicative state [Reactome] | 4,26E-05 | Psmb1;Psmd4;Psmd1;Psmb6;Psma6 |
|  | DNA Replication [Reactome] | 7,44E-05 | Psmb1;Psmd4;Rpa2;Psmd1;Psmb6;Rpa1;Psma6 |
|  | Intraflagellar transport [Reactome] | 9,33E-05 | Tubb1;Trip11;Dynll1;Tuba4a;Tuba1a;Tubb4b;Tuba1c;Tubb2a;Tubb6;Tubb2b;Dynll2;Tubb4a;Tubb3 |
|  | SYMPTOMATIC TIME POINT | | |
| SLOW C57 | **term** | **pvalue** | **Matched Genes** |
|  | Mus musculus biological processes [Reactome] | 0,000333757 | Hdac1;Sap18;Hdac3;Tgfb1;H2afz;Hist1h2bm;Lmnb1;Hspa2;H2afx;Stat5a;Stat5b;Hist1h4a; Hist1h4b; Hist1h4c; Hist1h4d; Hist1h4f; Hist1h4h; Hist1h4i; Hist1h4j; Hist1h4k; Hist1h4m; Hist2h4a; Hist4h4;Rps27a;Hist1h2ba;Hist1h3b; Hist1h3c; Hist1h3d; Hist1h3e; Hist1h3f; Hist2h3b; Hist2h3c1; Hist2h3c2;Cpt1a;Rbbp4;Rad21;Jak2;Hist2h2ab;Hist3h2bb;Smarca5;Smc1a |
|  | Negative epigenetic regulation of rRNA expression [Reactome] | 0,000515694 | Hdac1;Sap18;H2afz;Hist1h2bm;H2afx;Hist1h4a; Hist1h4b; Hist1h4c; Hist1h4d; Hist1h4f; Hist1h4h; Hist1h4i; Hist1h4j; Hist1h4k; Hist1h4m; Hist2h4a; Hist4h4;Hist1h2ba;Hist1h3b; Hist1h3c; Hist1h3d; Hist1h3e; Hist1h3f; Hist2h3b; Hist2h3c1; Hist2h3c2;Rbbp4;Hist2h2ab;Hist3h2bb;Smarca5 |
|  | NoRC negatively regulates rRNA expression [Reactome] | 0,000515694 | Hdac1;Sap18;H2afz;Hist1h2bm;H2afx;Hist1h4a; Hist1h4b; Hist1h4c; Hist1h4d; Hist1h4f; Hist1h4h; Hist1h4i; Hist1h4j; Hist1h4k; Hist1h4m; Hist2h4a; Hist4h4;Hist1h2ba;Hist1h3b; Hist1h3c; Hist1h3d; Hist1h3e; Hist1h3f; Hist2h3b; Hist2h3c1; Hist2h3c2;Rbbp4;Hist2h2ab;Hist3h2bb;Smarca5 |
|  | Epigenetic regulation of gene expression [Reactome] | 0,00055218 | Hdac1;Sap18;H2afz;Hist1h2bm;H2afx;Hist1h4a; Hist1h4b; Hist1h4c; Hist1h4d; Hist1h4f; Hist1h4h; Hist1h4i; Hist1h4j; Hist1h4k; Hist1h4m; Hist2h4a; Hist4h4;Hist1h2ba;Hist1h3b; Hist1h3c; Hist1h3d; Hist1h3e; Hist1h3f; Hist2h3b; Hist2h3c1; Hist2h3c2;Rbbp4;Hist2h2ab;Dek;Hist3h2bb;Smarca5;Ddx21 |
|  | Post-translational protein modification [Reactome] | 0,000611988 | Hgs;Usp24;Hist1h2al;Usp9x;Gm5422;Sec22b;Psmb1;Psmd4;Copb2;Cope;Calr;Ctsa;Proc;Rad23b;Ruvbl1;Copz1;Psmc6;Sumo1;Psme2;Vcp;Psmd1;Sptb;Sec31a;Hcfc1;Rad21;Dag1;Sptbn1;Hist2h2ab;Nop58;Galnt2;Tmed3;Snx3;Ppp6r1;Nup214;Nup54;Taf10;0610009B22Rik;Usp15;Psmd2;Sptbn4;Parp1;Derl1;Arfgap2;Tmed9;Sar1b;Psmd9;Smc1a;Lman1;Rab1b;Psmd12;Ngly1;Nup50;Uchl3;Usp14;Uso1;Hnrnpc |
|  | Translocation of GLUT4 to the plasma membrane [Reactome] | 0,000953483 | Tubb1;Sfn;Rab8a;Rab4a;Rab10;Ywhag;Ywhae;Ywhaz;Tuba4a;Tuba1a;Tubb4b;Tuba1c;Ywhah;Stx4;Exoc5;Tubal3;Stxbp3;Tubb2a;Lnpep;Myh9;Rab14;Tubb6;Tubb2b;Tubb4a;Tubb3;Tuba8;Prkab1 |
|  | Positive epigenetic regulation of rRNA expression [Reactome] | 0,001136616 | H2afz;Hist1h2bm;H2afx;Hist1h4a; Hist1h4b; Hist1h4c; Hist1h4d; Hist1h4f; Hist1h4h; Hist1h4i; Hist1h4j; Hist1h4k; Hist1h4m; Hist2h4a; Hist4h4;Hist1h2ba;Hist1h3b; Hist1h3c; Hist1h3d; Hist1h3e; Hist1h3f; Hist2h3b; Hist2h3c1; Hist2h3c2;Hist2h2ab;Dek;Hist3h2bb;Smarca5;Ddx21 |
|  | Meiotic synapsis [Reactome] | 0,002153946 | H2afz;Hist1h2bm;Lmnb1;Hspa2;H2afx;Hist1h4a; Hist1h4b; Hist1h4c; Hist1h4d; Hist1h4f; Hist1h4h; Hist1h4i; Hist1h4j; Hist1h4k; Hist1h4m; Hist2h4a; Hist4h4;Hist1h2ba;Hist1h3b; Hist1h3c; Hist1h3d; Hist1h3e; Hist1h3f; Hist2h3b; Hist2h3c1; Hist2h3c2;Rad21;Hist2h2ab;Hist3h2bb;Smc1a |
|  | Meiotic Synapsis [Reactome] | 0,002153946 | H2afz;Hist1h2bm;Lmnb1;Hspa2;H2afx;Hist1h4a; Hist1h4b; Hist1h4c; Hist1h4d; Hist1h4f; Hist1h4h; Hist1h4i; Hist1h4j; Hist1h4k; Hist1h4m; Hist2h4a; Hist4h4;Hist1h2ba;Hist1h3b; Hist1h3c; Hist1h3d; Hist1h3e; Hist1h3f; Hist2h3b; Hist2h3c1; Hist2h3c2;Rad21;Hist2h2ab;Hist3h2bb;Smc1a |
|  | CLEC7A (Dectin-1) signaling [Reactome] | 0,002197508 | Gm5422;Psmb1;Psmd4;Prkcd;Psmc6;Psme2;Psmd1;Psmd2;Psmd9;Psmd12 |
| FAST 129SV | **term** | **pvalue** | **Matched Genes** |
|  | Epigenetic regulation of gene expression [Reactome] | 8,91E-08 | Sf3b1;Hdac1;Sap18;H2afz;Hist1h2bm;H2afx;Hist1h4a; Hist1h4b; Hist1h4c; Hist1h4d; Hist1h4f; Hist1h4h; Hist1h4i; Hist1h4j; Hist1h4k; Hist1h4m; Hist2h4a; Hist4h4;Hist1h2ba;Hist1h3b; Hist1h3c; Hist1h3d; Hist1h3e; Hist1h3f; Hist2h3b; Hist2h3c1; Hist2h3c2;Rbbp4;Hist2h2ab;Dek;Hist3h2bb;Smarca5;Ddx21;H2afy |
|  | Negative epigenetic regulation of rRNA expression [Reactome] | 2,53E-06 | Hdac1;Sap18;H2afz;Hist1h2bm;H2afx;Hist1h4a; Hist1h4b; Hist1h4c; Hist1h4d; Hist1h4f; Hist1h4h; Hist1h4i; Hist1h4j; Hist1h4k; Hist1h4m; Hist2h4a; Hist4h4;Hist1h2ba;Hist1h3b; Hist1h3c; Hist1h3d; Hist1h3e; Hist1h3f; Hist2h3b; Hist2h3c1; Hist2h3c2;Rbbp4;Hist2h2ab;Hist3h2bb;Smarca5;H2afy |
|  | NoRC negatively regulates rRNA expression [Reactome] | 2,53E-06 | Hdac1;Sap18;H2afz;Hist1h2bm;H2afx;Hist1h4a; Hist1h4b; Hist1h4c; Hist1h4d; Hist1h4f; Hist1h4h; Hist1h4i; Hist1h4j; Hist1h4k; Hist1h4m; Hist2h4a; Hist4h4;Hist1h2ba;Hist1h3b; Hist1h3c; Hist1h3d; Hist1h3e; Hist1h3f; Hist2h3b; Hist2h3c1; Hist2h3c2;Rbbp4;Hist2h2ab;Hist3h2bb;Smarca5;H2afy |
|  | RHO GTPases activate PKNs [Reactome] | 3,83E-05 | Sfn;Ywhag;Ppp1cb;Ywhae;Rhob;Hist1h4a; Hist1h4b; Hist1h4c; Hist1h4d; Hist1h4f; Hist1h4h; Hist1h4i; Hist1h4j; Hist1h4k; Hist1h4m; Hist2h4a; Hist4h4;Ywhaz;Ywhah;Pkn1;Hist1h3b; Hist1h3c; Hist1h3d; Hist1h3e; Hist1h3f; Hist2h3b; Hist2h3c1; Hist2h3c2;Rhoc;4930544G11Rik |
|  | Mus musculus biological processes [Reactome] | 6,38E-05 | Hdac1;Sap18;Hdac3;H2afz;Hist1h2bm;Lmnb1;H2afx;Stat5a;Stat5b;Hist1h4a; Hist1h4b; Hist1h4c; Hist1h4d; Hist1h4f; Hist1h4h; Hist1h4i; Hist1h4j; Hist1h4k; Hist1h4m; Hist2h4a; Hist4h4;Rps27a;Hist1h2ba;Hist1h3b; Hist1h3c; Hist1h3d; Hist1h3e; Hist1h3f; Hist2h3b; Hist2h3c1; Hist2h3c2;Rbbp4;Jak2;Hist2h2ab;Hist3h2bb;Smarca5;Nampt;Smc1a;H2afy |
|  | Meiotic Recombination [Reactome] | 0,000200528 | H2afz;Hist1h2bm;H2afx;Hist1h4a; Hist1h4b; Hist1h4c; Hist1h4d; Hist1h4f; Hist1h4h; Hist1h4i; Hist1h4j; Hist1h4k; Hist1h4m; Hist2h4a; Hist4h4;Hist1h2ba;Hist1h3b; Hist1h3c; Hist1h3d; Hist1h3e; Hist1h3f; Hist2h3b; Hist2h3c1; Hist2h3c2;Hist2h2ab;Hist3h2bb;H2afy |
|  | Intraflagellar transport [Reactome] | 0,000357976 | Tubb1;Trip11;Tuba4a;Tuba1a;Tubb4b;Tuba1c;Tubb2a;Tubb6;Tubb2b;Tubb4a;Tubb3 |
|  | Striated Muscle Contraction [Reactome] | 0,000584558 | Actn3;Vim;Tmod1;Tpm2;Actc1;Acta1;Tpm4;Actn2 |
|  | Antigen Presentation: Folding, assembly and peptide loading of class I MHC [Reactome] | 0,000960069 | Sec24c;H2-Q10;H2-D1;H2-T23;Pdia3;Tap2;Sec31a;Bcap31;Sec24b;H2-Q4;Sar1b;Erap1 |
|  | Activation of anterior HOX genes in hindbrain development during early embryogenesis [Reactome] | 0,000965138 | Hdac3;H2afz;Hist1h2bm;H2afx;Hist1h4a; Hist1h4b; Hist1h4c; Hist1h4d; Hist1h4f; Hist1h4h; Hist1h4i; Hist1h4j; Hist1h4k; Hist1h4m; Hist2h4a; Hist4h4;Hist1h2ba;Hist1h3b; Hist1h3c; Hist1h3d; Hist1h3e; Hist1h3f; Hist2h3b; Hist2h3c1; Hist2h3c2;Rbbp4;Hist2h2ab;Hist3h2bb;H2afy |

**Supplementary Table 5**. **Proteomic data of the immunosenescence protein candidates selected for the re-test experiments**. The regulation of the selected candidates comparing Wild-type (WT) vs transgenic (ALS) for both FAST129 Sv and SLOW C57 genetic background is shown, together with the number of peptides and adjusted p-values (in bold statistically significant value for adjusted p value <0.3).

|  | PRE-SYMPTOMATIC | | | | |
| --- | --- | --- | --- | --- | --- |
| SLOW C57 | **Protein Group Accessions number** | **Protein Descriptions** | **Peptide**  **number** | **Log 2 Fold Change**  **ALS/ WT** | **Adjusted P.Value** |
|  | P16110 | Galectin-3 OS=Mus musculus GN=Lgals3 PE=1 SV=3 - [LEG3_MOUSE] | 2 | -0,110915255 | 0,816567881 |
|  | P08226 | Apolipoprotein E OS=Mus musculus GN=Apoe PE=1 SV=2 - [APOE_MOUSE] | 18 | -0,800338624 | **0,221708707** |
|  | O54890 | Integrin beta-3 OS=Mus musculus GN=Itgb3 PE=1 SV=2 - [ITB3_MOUSE] | 26 | 0,029099138 | 0,917060753 |
|  | P04202 | Transforming growth factor beta-1 OS=Mus musculus GN=Tgfb1 PE=1 SV=1 - [TGFB1_MOUSE] | 8 | -0,979091661 | **0,001218715** |
|  | Q00623 | Apolipoprotein A-I OS=Mus musculus GN=Apoa1 PE=1 SV=2 - [APOA1_MOUSE] | 46 | 0,302986728 | 0,319692108 |
| FAST 129 SV | **Protein Group Accessions number** | **Protein Descriptions** | **Peptide**  **number** | **Log 2 Fold Change**  **ALS/ WT** | **Adjusted P.Value** |
|  | P16110 | Galectin-3 OS=Mus musculus GN=Lgals3 PE=1 SV=3 - [LEG3_MOUSE] | 2 | -0,400349389 | 0,317256973 |
|  | P08226 | Apolipoprotein E OS=Mus musculus GN=Apoe PE=1 SV=2 - [APOE_MOUSE] | 18 | -0,343758451 | 0,629313258 |
|  | O54890 | Integrin beta-3 OS=Mus musculus GN=Itgb3 PE=1 SV=2 - [ITB3_MOUSE] | 26 | -0,37225969 | **0,083338057** |
|  | P04202 | Transforming growth factor beta-1 OS=Mus musculus GN=Tgfb1 PE=1 SV=1 - [TGFB1_MOUSE] | 8 | -0,563168219 | **0,044262524** |
|  | Q00623 | Apolipoprotein A-I OS=Mus musculus GN=Apoa1 PE=1 SV=2 - [APOA1_MOUSE] | 46 | 0,226267209 | 0,459240997 |
|  | SYMPTOMATIC | | | | |
| SLOW C57 | **Protein Group Accessions number** | **Protein Descriptions** | **Peptide**  **number** | **Log 2 Fold Change**  **ALS/ WT** | **Adjusted P.Value** |
|  | P16110 | Galectin-3 OS=Mus musculus GN=Lgals3 PE=1 SV=3 - [LEG3_MOUSE] | 2 | -0,752755801 | **0,059503017** |
|  | P08226 | Apolipoprotein E OS=Mus musculus GN=Apoe PE=1 SV=2 - [APOE_MOUSE] | 18 | -0,91349729 | **0,161181962** |
|  | O54890 | Integrin beta-3 OS=Mus musculus GN=Itgb3 PE=1 SV=2 - [ITB3_MOUSE] | 26 | 0,257137357 | 0,249593172 |
|  | P04202 | Transforming growth factor beta-1 OS=Mus musculus GN=Tgfb1 PE=1 SV=1 - [TGFB1_MOUSE] | 8 | -0,518463214 | **0,068921105** |
|  | Q00623 | Apolipoprotein A-I OS=Mus musculus GN=Apoa1 PE=1 SV=2 - [APOA1_MOUSE] | 46 | 0,243667042 | 0,432431606 |
| FAST 129 SV | **Protein Group Accessions number** | **Protein Descriptions** | **Peptide**  **number** | **Log 2 Fold Change**  **ALS/ WT** | **Adjusted P.Value** |
|  | P16110 | Galectin-3 OS=Mus musculus GN=Lgals3 PE=1 SV=3 - [LEG3_MOUSE] | 2 | 0,247078281 | 0,61237549 |
|  | P08226 | Apolipoprotein E OS=Mus musculus GN=Apoe PE=1 SV=2 - [APOE_MOUSE] | 18 | 0,373860757 | 0,644796653 |
|  | O54890 | Integrin beta-3 OS=Mus musculus GN=Itgb3 PE=1 SV=2 - [ITB3_MOUSE] | 26 | 0,245566746 | 0,317137826 |
|  | P04202 | Transforming growth factor beta-1 OS=Mus musculus GN=Tgfb1 PE=1 SV=1 - [TGFB1_MOUSE] | 8 | -0,132125794 | 0,722571746 |
|  | Q00623 | Apolipoprotein A-I OS=Mus musculus GN=Apoa1 PE=1 SV=2 - [APOA1_MOUSE] | 46 | 0,33148538 | 0,315995479 |
