# Supplementary material for: Tissue-enhanced plasma proteomic analysis for disease stratification in amyotrophic lateral sclerosis
Source: Mol Neurodegener. 2018 Nov 7;13:60. doi: 10.1186/s13024-018-0292-2 (PMC6223075; doi:10.1186/s13024-018-0292-2)
Supplement: Supplementary file 2 — Detailed protocols for Sample fractionation, LC-MS/MS analysis and peptide identification and quantification: methods are explained in more detail in this additional file. (PDF 14 kb) [file 13024_2018_292_MOESM2_ESM.pdf]

Additional file 1. Detailed protocols for Sample fractionation, LC-MS/MS analysis and peptide identification and quantification.

Strong Cation exchange (SCX) Fractionation

The SCX gradient was run with a flow rate of 2mL/min as follows: 0–10 minutes: 5mM KH2PO4 in 25% acetonitrile (pH 3 adjusted with H3PO4); 10–43 minutes: linear gradient from 0 to 250 mM KCl in 5 mM KH2PO4 in 25% acetonitrile (pH3). Based on the UV absorbance at 214 nm, ten fractions of one-minute during the gradient were selected and dried with a vacuum concentrator.

LC MS/MS method

Two solvents were used (solvent A: water with 0.1% formic acid; solvent B: acetonitrile with 0.1% formic acid) for elution. Peptides were separated at a flow rate of 200 nL/min and eluted from the column at 40°C, over a 240-minute gradient (from 3% to 30% solvent B in 220 min followed by an increase to 80% solvent B in 10mins which was held for a further 10 minutes). A shorter chromatography method with the same gradient was used for the mouse samples 180 min gradient (3% to 30% solvent B in 160 minutes, followed by an increase to 80% solvent B in 10 minutes, which was held for a further 10 minutes). A Top Speed Higher-energy Collisional Dissociation (HCD) method with a 3-second cycle was used with the following parameters: MS-Spray voltage: 2000V; ion transfer tube temperature: 275°C; detector: Orbitrap; scan range (m/z): 400-1400 (human) and 350-1500 (animal model); resolution: 120000; AGC target: $5x10^5$. MS/MS: HCD; Isolation mode: quadrupole; collision energy: 37%; detector: Orbitrap; Resolution: 60000; AGC target: $1x10^4$.

Peptide identification and quantification

The reporter ions quantifier node was set up to measure the raw intensity values for TMT® 10plex mono-isotopic ions (126, 127N, 127C, 128N, 128C, 129N, 129C, 130N, 130C, 131). The SEQUEST HT search engine was programmed to search for tryptic peptides (with up to two missed cleavages allowed), static modifications of carbamidomethyl (C), TMT6plex (K), and TMT6plex (N-Term) and dynamic modifications of deamidation (N/Q), oxidation (M), and phosphorylation (STY). Precursor mass tolerance was set to 20 ppm and fragment (b and y ions) mass tolerance to 0.02Da. Spectra were also searched using the PhosphoRS-3.1 (Taus, et al. 2011) (fragment mass tolerance of 20mmu, considering neutral loss peaks for CID and HCD) and Percolator nodes (The, et al. 2016). Raw intensity values were exported to tab delimited text files for processing and filtering using in-house software. Grouped protein results were exported to tab delimited “Multi-consensus.txt files”, filtered at 1% FDR (PSM level) and at least 1 x Rank 1 peptide per protein.

References

Taus, T., et al.
2011 Universal and confident phosphorylation site localization using phosphoRS. J Proteome Res 10(12):5354-62.

The, M., et al.
2016 Fast and Accurate Protein False Discovery Rates on Large-Scale Proteomics Data Sets with Percolator 3.0. J Am Soc Mass Spectrom 27(11):1719-1727.
